# Hetero‐trans‐β‐glucanase, an enzyme unique to *Equisetum* plants, functionalizes cellulose

**DOI:** 10.1111/tpj.12935

**Published:** 2015-08-25

**Authors:** Thomas J. Simmons, Kyle E. Mohler, Claire Holland, Florence Goubet, Lenka Franková, Douglas R. Houston, Andrew D. Hudson, Frank Meulewaeter, Stephen C. Fry

**Affiliations:** ^1^ The Edinburgh Cell Wall GroupInstitute of Molecular Plant SciencesThe University of EdinburghEdinburghEH9 3BFUK; ^2^ Bayer CropScience NVInnovation CenterTechnologiepark 389052GentBelgium; ^3^ Institute of Structural and Molecular BiologyThe University of EdinburghThe King's BuildingsEdinburghEH9 3JRUK; ^4^ Institute of Molecular Plant SciencesThe University of EdinburghEdinburghEH9 3BFUK; ^5^ Present address: Department of BiochemistryUniversity of CambridgeCambridgeCB2 1QWUK; ^6^ Present address: Department of Biochemistry175 South University Street, West LafayetteIN 47907USA

**Keywords:** plant cell wall, hemicelluloses, cellulose, transglycosylation, hetero‐transglycanase, enzyme evolution, *Equisetum*

## Abstract

Cell walls are metabolically active components of plant cells. They contain diverse enzymes, including transglycanases (endotransglycosylases), enzymes that ‘cut and paste’ certain structural polysaccharide molecules and thus potentially remodel the wall during growth and development. Known transglycanase activities modify several cell‐wall polysaccharides (xyloglucan, mannans, mixed‐linkage β‐glucan and xylans); however, no transglycanases were known to act on cellulose, the principal polysaccharide of biomass. We now report the discovery and characterization of hetero‐trans‐β‐glucanase (HTG), a transglycanase that targets cellulose, in horsetails (*Equisetum* spp., an early‐diverging genus of monilophytes). HTG is also remarkable in predominantly catalysing hetero‐transglycosylation: its preferred donor substrates (cellulose or mixed‐linkage β‐glucan) differ qualitatively from its acceptor substrate (xyloglucan). HTG thus generates stable cellulose–xyloglucan and mixed‐linkage β‐glucan–xyloglucan covalent bonds, and may therefore strengthen ageing *Equisetum* tissues by inter‐linking different structural polysaccharides of the cell wall. 3D modelling suggests that only three key amino acid substitutions (Trp → Pro, Gly → Ser and Arg → Leu) are responsible for the evolution of HTG's unique specificity from the better‐known xyloglucan‐acting homo‐transglycanases (xyloglucan endotransglucosylase/hydrolases; XTH). Among land plants, HTG appears to be confined to *Equisetum*, but its target polysaccharides are widespread, potentially offering opportunities for enhancing crop mechanical properties, such as wind resistance. In addition, by linking cellulose to xyloglucan fragments previously tagged with compounds such as dyes or indicators, HTG may be useful biotechnologically for manufacturing stably functionalized celluloses, thereby potentially offering a commercially valuable ‘green’ technology for industrially manipulating biomass.

## Significance Statement

Plants possess transglycanases, but none were known to act on cellulose, the principal biomass component; we now report the discovery in *Equisetum* of hetero‐trans‐β‐glucanase (HTG), which does target cellulose. Predominantly a hetero‐transglycanase, it cuts cellulose and attaches it to xyloglucan, making stable cellulose–xyloglucan covalent bonds and potentially affording a ‘green’ technology for manipulating cellulose post‐harvest and also in living crops. 3D‐Modelling suggests key amino‐acid substitutions responsible for the evolution of HTG from standard XTHs.

## Introduction

**Figure 1:**
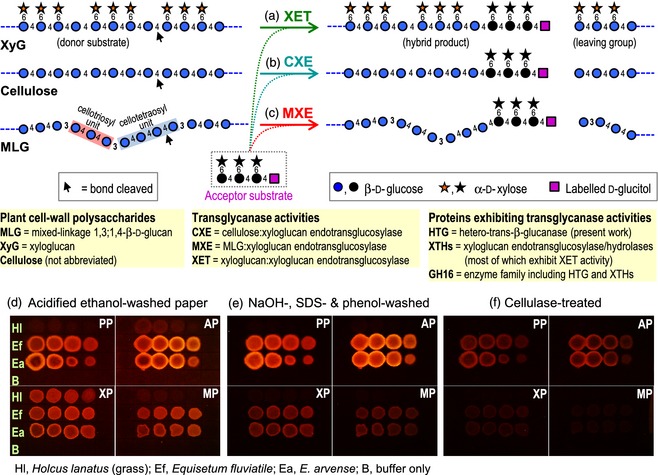
Three transglycanase activities of native *Equisetum* HTG. (a*–*c) Three reactions catalysed by HTG, with labelled xyloglucan heptasaccharide (XXXGol) as acceptor substrate (for explanation of XGO nomenclature, see Fry *et al*., 1993).  (d–f) Dot‐blot assays. Donor substrates: PP, plain paper (cellulose); AP, alkali‐washed paper; XP, xyloglucan‐impregnated paper; MP, MLG‐impregnated paper. Acceptor substrate: XXXG–sulforhodamine. Enzymes blotted comprise a dilution series (100, 50, 25, 12.5% v/v) of (NH_4_)_2_ SO_4_‐precipitated proteins from a grass (*Holcus lanatus*) or *Equisetum* (*E. fluviatile* and *E. arvense*). Polymeric reaction products were documented after sequential washing (d, e) and enzymolysis (f).

The morphology, size and strength of plant cells, and ultimately of whole plants, are determined by the cell wall, which is a complex fabric (Albersheim *et al*., 2011), some of whose structural polysaccharides, including xyloglucan and mixed‐linkage (1→3, 1→4)‐β‐d‐glucan (MLG), are re‐modelled *in vivo* by enzymes such as hydrolases, transglycanases and transglycosidases (Albersheim *et al*., 2011; Franková and Fry, 2013). Such remodelling is likely to adjust the biophysical properties of the cell wall, and thus the growth and strength of the plant. Known transglycanase activities include xyloglucan endotransglucosylase (XET) (Figure 1a), which grafts part of one xyloglucan chain onto another (Fry *et al*., 1992, 2008a; Nishitani and Tominaga, 1992; Rose *et al*., 2002; Eklöf and Brumer, 2010; Stratilová *et al*., 2010; Maris *et al*., 2011), trans‐β‐mannanase (Schröder *et al*., 2009) and trans‐β‐xylanase (Franková and Fry, 2011; Derba‐Maceluch *et al*., 2014). Recently, a unique hetero‐transglycanase activity, MLG:xyloglucan endotransglucosylase (MXE), was discovered (Fry *et al*., 2008a); this enzyme grafts a segment of MLG onto the non‐reducing terminus of a qualitatively different polysaccharide, xyloglucan (Figure 1c). Among land plants, appreciable extractable MXE activity (Fry *et al*., 2008a) and *in‐vivo* MXE action (Mohler *et al*., 2013) appear to be confined to *Equisetum* (horsetails).

*Equisetum* is a remarkable genus of ‘ferns’ (*sensu lato*; monilophytes) that diverged from its closest living relatives in the Upper Devonian period, more than 370 million years ago (Pryer *et al*., 2001; Knie *et al*., 2015). It is probably the most evolutionarily isolated of all land‐plant genera. Since its divergence from other land plants, it has acquired several unusual biochemical features, including the polysaccharide MLG (Fry *et al*., 2008b; Sørensen *et al*., 2008; Xue and Fry, 2012), MXE activity (Fry *et al*., 2008a; Mohler *et al*., 2013), and a high silica content (Kido *et al*., 2015).

Xyloglucan and MLG are hemicelluloses (Figure 1a,c), a proportion of which are thought to coat and/or penetrate the cellulosic microfibrils, and probably to tether adjacent microfibrils in plant cell walls (Albersheim *et al*., 2011; Park and Cosgrove, 2015). MLG is known to be abundant in many members of the Poales (grasses, cereals, reeds etc.; Smith and Harris, 1999) and *Equisetum* (Fry *et al*., 2008b; Sørensen *et al*., 2008), but not other land plants (Xue and Fry, 2012). [Some non‐*Equisetum* ferns and *Selaginella* have been reported to possess an MLG cross‐reacting epitope (Harholt *et al*., 2012; Leroux *et al*., 2015). However, the monoclonal antibody BS‐400‐3 (formerly named BG1) used is not completely specific for MLG: it also weakly detects cellopentaose (Meikle *et al*., 1994). The reported labelling of pteridophyte specimens with this antibody may therefore indicate the presence of either MLG or some other polysaccharide with a run of (1→4)‐β‐glucose residues. Neither Leroux *et al*. (2015) nor Harholt *et al*. (2012) provided chemical evidence for MLG. Thus further work is required before the presence of MLG in non‐*Equisetum* pteridophytes is proven]. Xyloglucan and cellulose, in contrast to MLG, are ubiquitous components of land plants (Albersheim *et al*., 2011).

Polysaccharide‐modifying enzymes may be valuable for manipulating cell‐wall architecture. Transglycanases cleave one polysaccharide chain (the donor substrate) and then graft a length thereof onto another poly‐ or oligosaccharide (the acceptor substrate) (Figure 1a–c) (Franková and Fry, 2013). XET (Figure 1a) is termed a homo‐transglycanase because the donor and acceptor are both xyloglucan. Plant enzymes possessing predominantly XET activity (Figure 1a) and/or the corresponding endohydrolase activity are termed XTHs (xyloglucan endotransglucosylase/hydrolases) (Rose *et al*., 2002). Remarkably, the *Equisetum* enzyme exhibits MXE activity (Figure 1c) as its major activity, and is thus a predominant hetero‐transglycanase (donor MLG; acceptor xyloglucan) (Fry *et al*., 2008a; Mohler *et al*., 2013). All other known plant hetero‐transglycanase activities are considered to be side reactions of predominant homo‐transglycanases (Hrmova *et al*., 2007; Stratilová *et al*., 2010), although laminarin:chitin hetero‐transglycanase activity has been observed in the yeast *Saccharomyces cerevisiae* (Cabib *et al*., 2008).

We have purified and characterized the protein responsible for MXE, an activity that has been demonstrated previously only in total *Equisetum* plant extracts (Fry *et al*., 2008a). We identified the corresponding gene and showed that the encoded protein acts not only on MLG and xyloglucan but also, surprisingly, on cellulose, the world's most abundant organic material (Teeri *et al*., 2007), thus exhibiting cellulose:xyloglucan endotransglucosylase (CXE) activity (Figure 1b). This promiscuity led us to re‐name the protein hetero‐trans‐β‐glucanase (HTG). The potential biological, evolutionary and biotechnological significance of HTG is discussed.

## Results

### CXE activity of extracted *Equisetum* protein

*Equisetum* extracts exhibited a high MXE:XET ratio (Figure S1c). Unexpectedly, they also possessed an enzyme activity that we had not previously observed. We refer to this activity as CXE (Figure 1b), using cellulose as the donor substrate and labelled xyloglucan oligosaccharides (XGOs, e.g. the heptasaccharide XXXGol), fluorescently tagged with sulforhodamine or radioactively tagged with ^3^H, as the acceptor substrate, creating cellulose–XGO bonds. Previous work reporting CXE‐like activity used soluble derivatives [hydroxyethylcellulose and cellulose sulfate; the latter is produced by esterifying cellulose with H_2_SO_4_ (Fehling, 1845; Whistler and Spencer, 1963) and is sometimes referred to as ‘H_2_SO_4_‐swollen cellulose’] rather than cellulose itself (Hrmova *et al*., 2007; Kosík *et al*., 2010). In contrast, the principal donor substrate tested by us was Whatman filter paper, which comprises highly purified, insoluble cotton cellulose containing only traces of xylans, mannans and arabinogalactans.

Clear evidence for CXE activity in *Equisetum* extracts (*E. fluviatile* and *E. arvense*) is shown in Figure 1(e) (rows Ef and Ea), on plain paper or paper pre‐treated with alkali (‘PP’ and ‘AP’), on both of which these extracts produced an alkali‐stable fluorescent spot of cellulose–XGO–sulforhodamine. The resistance of the fluorescent product to 6 m NaOH indicates covalent cellulose–XGO bonding. Attachment of trace non‐cellulosic polysaccharides of paper to the XGO would have yielded alkali‐soluble products such as xyloglucan–XGO. A comparable extract of an angiosperm, the grass *Holcus lanatus*, exhibited negligible CXE activity (row Hl), as did pure buffer (row B) (Figure 1e).

The grass extract was included as a control, and was expected to contain XET but not MXE or CXE activity. In accordance with this, the grass extract generated a fluorescent polymer (xyloglucan–XGO–sulforhodamine) only if the paper had been impregnated with xyloglucan (Figure 1d, ‘XP’ paper). The identity of this ethanol‐insoluble fluorescent polymer as xyloglucan–XGO–sulforhodamine is confirmed by its solubilization on washing in 6 m NaOH (Figure 1e, ‘XP’ paper). The absence of MXE activity in the grass extract is confirmed by the lack of an MLG‐dependent fluorescent product (Figure 1d, compare 'MP’ with ‘PP’ and ‘AP’).

The *Equisetum* extracts produced fluorescent polymers on all four papers (Figure 1d). Based on this result alone, it is not possible to discriminate between CXE, MXE and XET activities. However, washing the papers in 6 m NaOH removed the MXE and XET products (MLG–XGO–sulforhodamine and xyloglucan–XGO–sulforhodamine respectively). Thus the fluorescence in Figure 1(d) minus that in Figure 1(e) equates to the MXE or XET product, and fluorescence in Figure 1(e) represents the CXE product. On this basis, we conclude that the *Equisetum* extracts possess all three activities, and, judged by the fluorescence, CXE activity exceeded XET and MXE activity. Comparison of Figure 1(d) with Figure 1(e) also indicated that the xyloglucan or MLG present in the paper partially competes with cellulose for utilization by CXE activity, suggesting that MXE, XET and CXE activities are all attributable to a single HTG protein. A proportion of the *Equisetum* CXE product even resisted cellulase treatment (Figure 1f), implying that the cellulose–XGO was integrated within the paper fibres.

Filter paper was somewhat more effective as a donor substrate if pre‐treated with alkali (Figure 1; ‘AP’), which converts cellulose I to the anti‐parallel cellulose II allomorph (Kroon‐Batenburg and Kroon, 1997), and simultaneously removes contaminating non‐cellulosic polysaccharides.

Further evidence of the cellulose–XGO nature of the reaction product was provided by determination of its solubility properties (Table S1). The radioactively labelled CXE product (cellulose–[^3^H]XGO) readily dissolved in LiCl/dimethylacetamide, a cellulose solvent, and then re‐precipitated when diluted into 6 m NaOH (an excellent solvent for hemicelluloses but not cellulose), confirming that the material obtained was not a hemicellulose–[^3^H]XGO contaminant. Our approach clearly demonstrates covalent attachment of cellulose to an XGO. As the (labelled) reducing terminus of the oligosaccharide remains present, the only plausible explanation is the reaction shown in Figure 1(b).

### Purification and partial characterization of native *Equisetum* HTG

All three activities (MXE, XET, CXE) approximately co‐migrated during native gel electrophoresis (Figure S1d), supporting the idea of a single promiscuous HTG protein. The slight mis‐match between the three bands was probably due to the difficulty of manually aligning three separate strips of gel on the different test papers.

**Figure 2:**
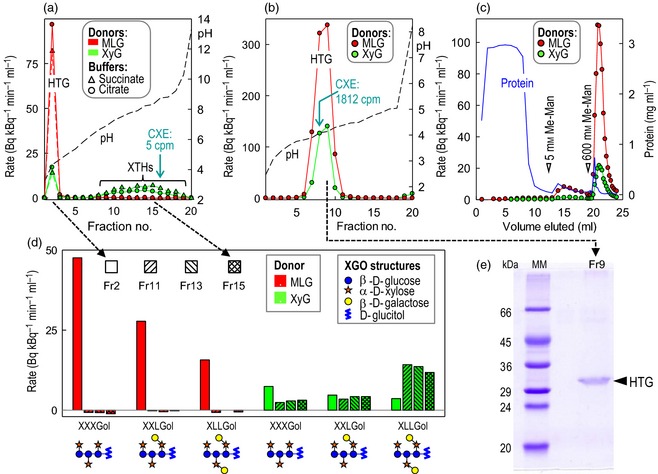
Characterization of native *Equisetum* HTG.  (a, b) MXE activity (red) and XET activity (green) of protein fractions obtained by preparative isoelectric focusing (IEF) from *E. fluviatile* shoots in May (a) or September (b); selected fractions were also assayed for CXE activity (values indicated in cyan). (c) Lectin affinity chromatography of HTG; an MXE‐active fraction from Bio‐Gel P‐100 (Figure S1b) was applied to concanavalin A–Sepharose and eluted with methyl α‐mannopyranoside. The MXE activity (red) and XET activity (green) co‐eluted. (d) Acceptor substrate specificity of IEF‐resolved MXE and XET activities. Four fractions from (a) were assayed with two donor and three acceptor substrates. (e) SDS–PAGE of IEF‐purified HTG [fraction 9 from (b)].

We further characterized the native HTG protein from *Equisetum* by several complementary approaches, in each case monitoring the protein on the basis of its (inseparable) MXE and XET activities. First, HTG pelleted in 20%‐saturated (NH_4_)_2_SO_4_ (Figure S1c), implying a relatively hydrophobic protein. Second, HTG eluted from a cation‐exchange chromatography column at pH 4.1 (Figure S1a), indicating a highly acidic protein. Third, on gel‐permeation chromatography, HTG eluted with an apparent *M*_r_ of approximately 10^4^ (Figure S1b), which, in the light of the SDS–PAGE results (see below), suggests an unusual affinity for the polyacrylamide matrix of Bio‐Gel P‐100. Fourth, by isoelectric focusing (IEF), HTG was confirmed to be highly acidic (isoelectric point, pI, of approximately 4.1; Figure 2a,b), whereas standard *Equisetum* XTHs (possessing XET activity but negligible MXE or CXE activity) had pI values in the range 6.6–9.0 (Figure 2a). Finally, HTG was found to bind to a concanavalin A affinity chromatography column (Figure 2c), indicating the presence of *N‐*glycosylation.

Of the MXE‐active fractions tested, all were also shown to be CXE‐active (e.g. Figure 2b).

After HTG had been partially purified by IEF, it ran as a 31‐kDa protein on SDS–PAGE (Figure 2e).

Table S2 shows a representative balance‐sheet for HTG purification from *Equisetum*. The purified enzyme was quantified and assayed for MXE activity at optimal substrate concentrations. The catalytic efficiency (*k*_cat_/*K*_M_) was very high compared with conventional XTHs (Table 1). Purified HTG was also unusual in preferring non‐galactosylated oligosaccharides as acceptor substrates (for both MXE and XET activities; Figure 2d), whereas the approximately neutral *Equisetum* XTHs preferred galactosylated XGOs, as do most dicot XTHs (Fry *et al*., 1992, 2008a; Steele and Fry, 2000; Eklöf and Brumer, 2010; Maris *et al*., 2011).

**Table 1:** Turnover number (*k*_cat_) and catalytic efficiency (*k*_cat_/*K*_M_) of *Equisetum* HTG compared with XTHs having predominantly XET activity

Enzyme	Donor substrate (mg ml^−1^)	Acceptor substrate	*k*_cat_ (min^−1^)	*K*_M_ (μm) for the indicated acceptor substrate			*k*_cat_/*K*_M_ (mm ^−1^ min^−1^)
				XET activity	MXE activity	CXE activity	
HTG ex *Pichia*	5.0^a^	XXXG		3.9 ± 0.5	1.4 ± 0.1		
HTG ex *Pichia*	5.0^a^	XXXGol	0.266^e^	3.4 ± 0.4	0.52 ± 0.06		510^e^
HTG ex *Pichia*	1.5^a^	XXXGol		0.77 ± 0.13	0.85 ± 0.07		
HTG ex *Pichia*	0.5^a^	XXXGol		0.91 ± 0.04	0.58 ± 0.14		
HTG ex *Pichia*	Approximately 900^b^	XXXGol				2.7 ± 0.5	
Native *Equisetum* HTG^c^	MLG, 5.0	XXXGol	0.086, 0.069		[0.52]^d^		170, 130
HTG ex *Pichia*	MLG, 5.0	XXXG	0.270		[0.52]^d^		520
Poplar XET^f^	XyG	Glc_8_‐based XGO mixture	4.8	400			12
Barley HvXET5^g^	XyG	XXXGol	0.34, 0.38	69			4.9, 5.6

a Soluble donor substrate: tamarind xyloglucan or barley MLG, as appropriate.

b Insoluble donor substrate: 18.6 mg cellulose (alkali‐washed paper) + 20 μl total aqueous solution.

c Data for two independent preparations of *Equisetum fluviatile* HTG.

d Assumed.

e The value given is for MXE activity.

f Data from Baumann *et al*. (2007).

g Data from the erratum to Hrmova *et al*. (2007).

### Sequencing *Equisetum* HTG

Tryptic digestion of *E. fluviatile* (Ef) HTG purified by IEF followed by SDS–PAGE yielded major oligopeptides with *m*/*z* values of approximately 963.43 and 1548.59 [(M + 1)^+^; Figure S2a]. On LC–MS/MS, these oligopeptides gave fragmentation mass spectra matching the predicted spectra of two inferred tryptic peptides (LYPNGFPR and SFPNNEAIGVPYLK) from among the *in‐silico* translation products of an Ef transcriptome (Figure S2b,c).

We obtained a full‐length sequence of *EfHTG* by performing 5′‐ and 3′‐RACE on *E. fluviatile* cDNA. The *in‐silico* translation product (non‐glycosylated and minus the predicted signal sequence; Figure S3a) has predicted *M*_r_ and pI values (29 500 and 4.66, respectively) that are comparable to those of native HTG. The sequence places HTG within XTH group I/II (Figure S4), all other members of which possess XET activity but negligible MXE and CXE activity (Rose *et al*., 2002; Hrmova *et al*., 2007; Eklöf and Brumer, 2010; Stratilová *et al*., 2010; Maris *et al*., 2011).

*HTG*‐like genes occur in other *Equisetum* species (Figure S3b), but have not been detected in other land plants, agreeing with the finding that MXE activity is apparently confined to *Equisetum* (Fry *et al*., 2008a; Mohler *et al*., 2013). The predicted protein has 28 acidic residues (the mean number for all Arabidopsis XTHs is 28.7) and 21 basic residues (the mean number for all Arabidopsis XTHs is 36.6); thus HTG's acidity is due to a lack of basic residues. HTG has four conserved cysteine residues, which are typical of XTHs, and one predicted *N‐*glycosylation site.

### Enzymic activities of heterologously produced *Equisetum* HTG

**Figure 3:**
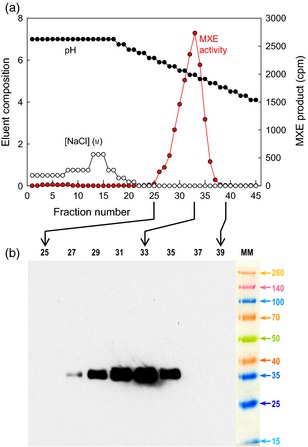
Nickel affinity chromatography of *Pichia*‐produced HTG. (a) MXE‐active fractions were eluted from a nickel column by a decreasing pH gradient. (b) Selected fractions were then subjected to SDS–PAGE, blotted, and detected with an anti‐His tag antibody. ‘MM’ represents a polypeptide marker mixture (*M*_r_ values × 10^−3^ are indicated).

**Figure 4:**
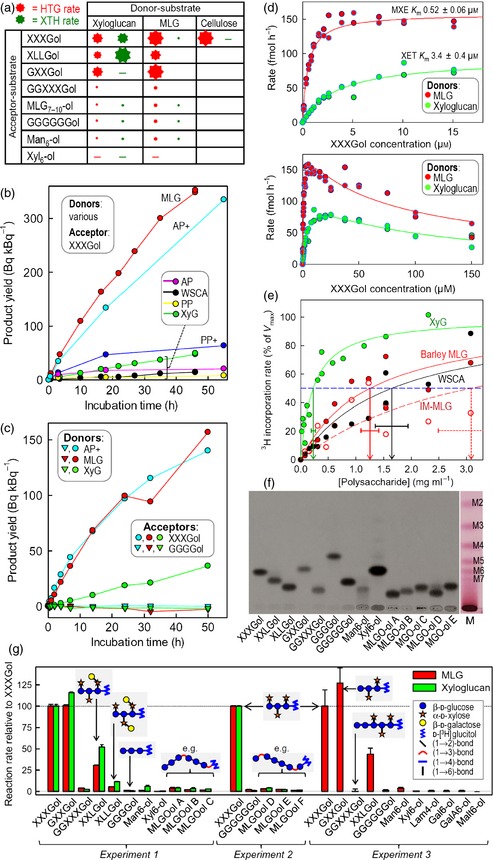
Enzymology of *Pichia*‐produced HTG. (a) Simplified summary of reactions catalysed by HTG (red) and typical XTHs (green). The size of the star indicates relative rate; a minus symbol indicates a negligible reaction; blank spaces indicate combinations that were not tested. (b) HTG‐catalysed transglycosylations with [^3^H]XXXGol as acceptor substrate and various donor substrates (WSCA, water‐soluble cellulose acetate; PP, plain paper; AP, alkali‐treated paper). Plus symbols indicate that BSA was added. (c) Transglycosylation with [^3^H]XXXGol but not [^3^H]cellotetraitol (GGGGol). (d) Affinity of HTG for XXXGol. (e) Affinity for donor polysaccharides. Vertical arrows indicate mean *K*_M_ ± SE. IM‐MLG, Iceland moss MLG (‘lichenan’). The *V*_max_ values (Bq kBq^−1^ h^−1^) were: xyloglucan, 0.626 ± 0.057; barley‐MLG, 7.59 ± 0.60; WSCA, 0.29 ± 0.03; IM‐MLG, 0.098 ± 0.014. (f) Thin‐layer chromatography of ^3^H‐oligosaccharides. Main image, fluorogram; M, malto‐oligosaccharide ladder. ‘MLGO‐ols’ are hepta‐ to decasaccharides from barley MLG partially digested with lichenase (MLGO‐ols A–C) or cellulase (MLGO‐ols D–E). Other abbreviations are defined in Experimental procedures. (g) HTG‐catalysed transglycosylation with MLG or xyloglucan as donor substrate and various ^3^H‐oligosaccharides as potential acceptors. Experiments 2 and 3 used Ni column‐purified HTG.

When *HTG* (without its signal‐encoding sequence) was expressed in the yeast *Pichia pastoris*, the EfHTG protein, detected by western blotting at approximately 36 kDa (Figure 3), had catalytic properties (Figure 4) that were notably different from those of typical XTHs. HTG had an MXE:XET activity ratio of approximately 7:1, whereas the ratio for Poaceae XTHs is typically approximately 0.002:1 (Hrmova *et al*., 2007; Fry *et al*., 2008a). Iceland moss *(Cetraria islandica)* MLG, largely comprising cellotriosyl repeat units (Figure 1c), was a poor donor substrate. Thus HTG probably recognizes cellotetraosyl repeat units, which occur in MLG from barley (*Hordeum vulgare*) and predominate in *Equisetum* MLG (Fry *et al*., 2008b; Sørensen *et al*., 2008; Xue and Fry, 2012). This, together with our previous characterization of MXE products (Mohler *et al*., 2013), indicates that the reaction catalysed when HTG acts on MLG plus an XGO is

…G4G4G3**G4G4G4G**3G4G4G… + [^3^H]XXXGol →

…G4G4G3**G4G4G4**‐[^3^H]XXXGol + **G**3G4G4G…

where G represents β‐d‐glucose, 3 represents a (1→3) bond, and 4 represents a (1→4) bond.

Cellohexaose, the largest water‐soluble fragment of cellulose, was not a donor substrate (Figure S5), suggesting that the enzyme needs to recognize a larger stretch of its donor substrate. In addition, water‐soluble cellulose acetate was a poor donor substrate (Figure 4b). However, *Pichia*‐produced HTG (like native *Equisetum* HTG) had remarkable CXE activity on the cellulose of filter paper, especially when this donor substrate had been pre‐treated with alkali and when the HTG was supplemented with bovine serum albumin (BSA) as an inert ‘carrier protein’ (Figure 4b). BSA had little effect when the donor substrates were water‐soluble, i.e. when MXE and XET were assayed (Figure S6).

**Figure 5:**
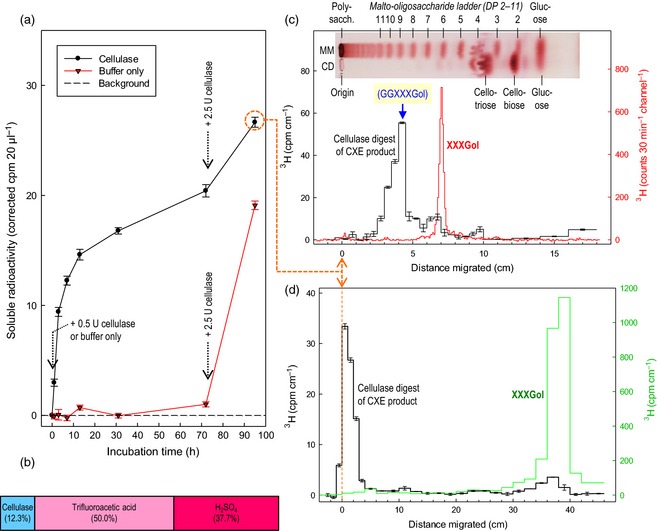
Characterizing the CXE product by cellulase digestion. (a) Samples (28–31 mg) of CXE product (formed by *Pichia*‐produced *Equisetum* HTG acting on alkali‐treated filter paper) were incubated in buffer (with antimicrobial chlorobutanol) and, where indicated, xyloglucan‐inactive cellulase. Solubilized radioactivity was assayed. (b) Proportion of ^3^H in the CXE product solubilized sequentially by cellulase [as in (a)], by 2 m trifluoroacetic acid (110°C for 2 h), and by the Saeman H_2_ SO_4_ hydrolysis method (Saeman *et al*., 1963). (c) A portion of the 95 h digest [orange circle in (a)] was analysed by TLC (three ascents); strips were assayed for ^3^H (±SE of replicate counts) by scintillation counting (black histogram). Pure [^3^H]XXXGol, chromatographed on the same plate, was monitored on a LabLogic ( www.lablogic.com/) AR2000 plate scanner (red histogram). Inset: malto‐oligosaccharide marker mixture (MM) and a further portion of the cellulase digest (CD), run on the same plate as the radioactive samples and stained with thymol/H_2_ SO_4_; the image is accurately aligned with the *x* axis. Blue arrow: position of authentic GGXXXGol (Mohler *et al*., 2013) relative to maltononaose. (d) As (c) but paper chromatographic analysis; 1‐ or 2‐cm strips of the paper were assayed for ^3^H by scintillation counting. Note that the cellulase digestion product does migrate (albeit slightly) in this chromatography solvent and is therefore oligomeric, not polymeric: the sample was loaded on the chromatogram as a 1‐cm‐diameter spot centred at ‘0 cm’; undigested polysaccharide would have been distributed equally between the ‘−1 to 0 cm’ strip and the ‘0 to +1 cm’ strip, and none would have been present in the ‘+1 to +2 cm’ strip.

As expected, the CXE product (cellulose–[^3^H]XXXGol) was partially digested by xyloglucan‐inactive cellulase to release water‐soluble ^3^H (Figure 5a). The cellulase did not completely digest the filter paper, even though paper is almost pure cellulose, because no synergistic enzyme such as a cellobiohydrolase was added. Nevertheless, the cellulase released stainable cello‐oligosaccharides from the filter paper (Figure 5c, inset), and 12.3% of the radioactivity was slowly released into solution, indicating gradual digestion of some of the peripheral cellulose chains of the paper fibres (Figure 5a,b). A buffer‐only control released no ^3^H (Figure 5a). These observations confirm that the HTG had indeed formed a covalent cellulose–[^3^H]XXXGol bond. A further 50% of the initial ^3^H in the CXE product was subsequently solubilized by trifluoroacetic acid (TFA), and the remaining 37.7% by H_2_SO_4_ (Figure 5b). The resistance of some of the cellulose–[^3^H]XXXGol to cellulase and even TFA confirms the conclusion from the results shown in Figure 1(f): some of the cellulose–XGO was well integrated within the paper fibres and thus shielded from cellulase.

We have previously shown (Mohler *et al*., 2013) that MLG–[^3^H]XXXGol, a polymeric product of MXE activity, may be hydrolysed by lichenase to yield a radioactive oligosaccharide comprising the original XGO with two additional glucose residues:

…G3G4G4G4G3G4G4G‐[^3^H]XXXGol [lichenase] → MLG oligosaccharides + G4G–[^3^H]XXXGol

Applying a similar strategy but with xyloglucan‐inactive cellulase instead of lichenase, we expect to obtain comparable radiolabelled oligosaccharides from cellulose–[^3^H]XXXGol. As predicted, the cellulase‐solubilized ^3^H had a mobility on TLC (Figure 5c) that was identical to that of G4G–[^3^H]XXXGol, which is its most likely identity, generated thus:

…G4G4G4G4G4G4G4G–[^3^H]XXXGol—[cellulase]→ cello‐oligosaccharides + G4G–[^3^H]XXXGol

On paper chromatography, the same oligomeric product had a mobility much lower than that of XXXGol (Figure 5d), indicating that the cellobiose tail (G4G) confers a strong ability to hydrogen bond to paper in the chromatography solvent used (Figure 5a). The observations confirm the previous conclusion, reached from the results in Figure 1(d–f) and Table S1, that HTG catalyses the CXE reaction shown in Figure 1(b).

**Figure 6:**
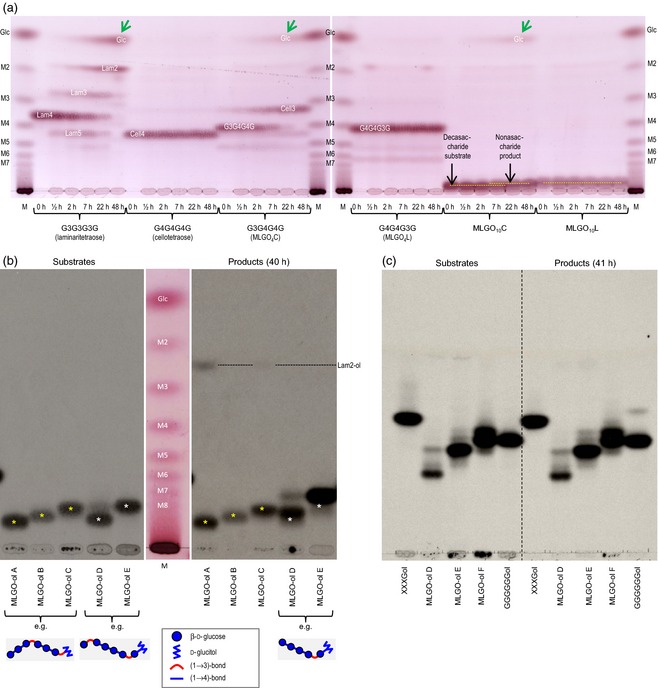
Characterizing MLG oligosaccharides (MLGOs) and their stability in the presence of purified HTG. (a) *Pichia* culture filtrate as a tool to probe non‐reducing termini. Non‐radioactive oligosaccharides were incubated with culture filtrate for 0–48 h, then analysed by TLC (one ascent). MLGO_*n*_C and MLGO_*n*_L are oligosaccharides with degree of polymerization *n*, produced by digestion of MLG with cellulase (C) or lichenase (L); M2–M7, malto‐oligosaccharide ladder. Oligosaccharides with a (1→3) bond at the non‐reducing end lost a glucose residue, giving a faster‐migrating oligosaccharide plus free glucose (indicated by green arrows), indicating (1→3)‐β‐glucosidase action; those with a (1→4) bond at the non‐reducing end were stable. The figure shows two TLC plates (left and right), which have been aligned as accurately as possible. (b) Crude *Pichia* culture filtrate attacks [^3^H]MLGO‐ols with a (1→3) bond at the non‐reducing end. Fluorographs of a thin‐layer chromatogram (with single ascent) of substrates (left) and products after 40 h incubation with *Pichia* filtrate (right). MLGOs were produced by incomplete digestion of MLG with lichenase (A–C) or cellulase (D, E); estimated degrees of polymerization are 10 (A), 9 (B), 8 (C), 10 (D) and 8 (E). A–C were essentially stable; D and E almost completely disappeared and were replaced by a ^3^H‐labelled product that is one degree of polymerization smaller owing to exo‐(1→3)‐β‐glucosidase action (e.g. G3G4G4G4G3G4G4G4Gol → Glc + G4G4G4G3G4G4G4Gol). The precise sequence of mid‐chain (1→3) and (1→4) bonds was not defined. (c) Nickel affinity‐purified EfHTG (ex *Pichia*) does not hydrolyse ^3^H‐oligosaccharides with a (1→3) bond at the non‐reducing end. Left, substrates; right, products after 41 h incubation. Fluorograph of a TLC (three ascents). The degrees of polymerization of MLGO‐ols D and E are 10 and 8, respectively. Cellulase‐generated MLGO‐ol F has estimated degree of polymerization of 7. This observation confirms that the inability of MLGO‐ols to act as acceptor substrates for HTG is not simply due to hydrolysis (instability) of these acceptor substrates.

Of 19 radiolabelled oligosaccharides tested, XGOs were by far the best acceptor substrates (Figure 4f,g). MLG‐derived oligosaccharides [regardless of whether the non‐reducing terminal glucose residue was (1→3)‐ or (1→4)‐linked] (Figure 6) and cello‐oligosaccharides were weak acceptor substrates. Thus, while HTG accommodates both MLG and cellulose as donor substrates, MLG‐ and cello‐oligosaccharides cannot readily re‐enter the enzyme's active site as acceptor substrates. Importantly, HTG possesses only slight MLG:MLG and cellulose:cellulose homo‐transglucanase activities.

Non‐galactosylated XGOs were the preferred acceptor substrates (Figure 4g). Surprisingly, GXXGol was highly effective, again distinguishing HTG from conventional XTHs, which require xylose at subsite +1 (for definition see Table S3) (Saura‐Valls *et al*., 2006). HTG does require xylose at subsite +2, as demonstrated by its inability to utilize GGXXXGol, cello‐oligosaccharides and MLG‐oligosaccharides as acceptor substrates.

HTG had a much higher affinity for XGOs (*K*_M_ < 3.4 μm XXXGol; Figure 4d) than do conventional XTHs [*K*_M_ vakues of approximately 50–200 μm XXXGol (Steele and Fry, 2000; Rose *et al*., 2002; Hrmova *et al*., 2007) or 1.1 mm XXXG–8‐aminonaphthalene‐1,3,6‐trisulfonate (Saura‐Valls *et al*., 2006)]. The *range* of apparent *K*_M_ values that we observed for HTG (0.52–3.4 μm XXXGol) probably arose because non‐radioactive xyloglucan, unlike MLG, competes with [^3^H]XXXGol as an acceptor substrate. The range of acceptor substrate *K*_M_ values narrowed to become consistently <1 μm in the presence of lower donor substrate concentrations (Table 1). High XXXGol concentrations inhibited *Pichia*‐produced EfHTG (Figure 4d), as observed in *Equisetum* extracts (Fry *et al*., 2008a), possibly because this oligosaccharide competed with the polysaccharides for binding to the enzyme's negative subsites without being able to serve as a donor substrate.

### Homology modelling of HTG

**Figure 7:**
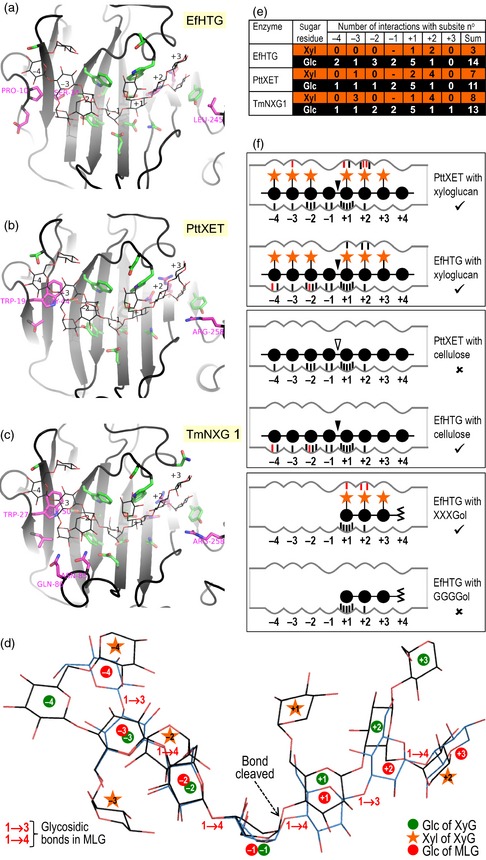
Modelled interaction of substrates with the active site in HTG and classical XTHs. (a–c) Substrate binding pocket of EfHTG and two XTHs (PttXET and TmNXG1). Grey ribbon, protein backbone; line drawing, XXXGXXG; sticks with green carbons, side chains lining the active‐site cleft; magenta carbons, residues that are functionally non‐conserved between HTG and both PttXET and TmNXG1. Residues that differ in their xyloglucan interactions between EfHTG (or its homologues in other *Equisetum* spp.) and both PttXET and TmNXG1 (Table S3) are numbered. (d) Two substrates modelled in EfHTG. Black carbons, XXXGXXG (a xyloglucan oligosaccharide); blue carbons, G3G4G4G4G3G4G (an MLG oligosaccharide). (e) Cumulative enzyme–sugar interactions (see Table S3). (f) Diagram of EfHTG and PttXET showing strong interaction (tick symbol) or weak interaction (cross) with polysaccharides and potential acceptor substrates; black and white arrowheads indicate bonds that are actually or potentially cleaved. Predicted interactions that are not present in a comparator are shown in red.

To explore the basis of HTG's unique substrate specificity, we modelled it alongside the established 3D structures of two angiosperm XTHs (Johansson *et al*., 2004; Mark *et al*., 2009) with the dodecasaccharide XXXGXXG in subsites −4 to +3 (Figure 7). Strikingly, HTG has no predicted interactions with negative subsite xyloses, but has additional interactions with negative subsite glucoses (Table S3), matching its ability to utilize cellulose as a donor substrate. All three proteins have multiple predicted interactions with the +2 subsite xylose, but HTG lacks one such interaction with the +1 subsite xylose, consistent with HTG's ability to utilize GXXGol but not GGXXXGol as an acceptor substrate (Figure 7e,f).

Bacterial lichenase (PDB ID 1U0A) (Gaiser *et al*., 2006) accommodates a G4G4G3G block of its substrate, MLG, in subsites −4 to −1, but G4G4G3G does not fit in the equivalent position of HTG. Instead, and consistent with our conclusion that the MXE product is …G4G4G3G4G4G4‐XXXGol, we propose that G3G4G4G fits into HTG subsites −4 to −1, with the −4 glucose positioned approximately where the −4 xylose of xyloglucan would lie (Figure 7d). The interactions that XTHs make with the glucose residues of xyloglucan are shared with those of MLG in subsites −3 to +1 of HTG; therefore HTG makes more interactions with MLG than do XTHs, consistent with the near inactivity of XTHs on MLG.

## Discussion

HTG is a highly unusual enzyme, mainly exhibiting hetero‐transglucanase activity with cellulose or MLG as preferred donor substrates and XGOs as acceptor substrates (i.e. CXE and MXE activity; Figure 1b,c). It also has limited XET (a homo‐transglucanase) activity, but negligible cellulose:cellulose or MLG:MLG homo‐transglucanase activities. Given the intermediary nature of the structure of cellulose between xyloglucan and MLG (xyloglucan = cellulose with side chains; MLG = cellulose with backbone linkage variation; Figure 1a–c), it is perhaps unsurprising that an enzyme capable of catalysing both MXE and XET activities is able to utilize cellulose as its donor substrate, exhibiting CXE activity. The lax donor substrate specificity of HTG somewhat resembles the substrate specificity of an angiosperm β‐glucanase (*Populus trichocarpa* endoglucanase 16; PtEG16) that hydrolyses water‐soluble cello‐oligosaccharides, MLG, xyloglucan and artificial cellulose derivatives (its ability to hydrolyse cellulose has not been reported) (Eklöf *et al*., 2013). However, there are major differences between PtEG16 and HTG: only the latter catalyses detectable hetero‐transglycosylation, and HTG recognizes a lengthy section of the cellulose chain such that water‐soluble cello‐oligosaccharides are not effective donor substrates. Furthermore, PtEG16 and HTG are only distantly related based on their sequences (maximum 31% amino acid identity). The MXE:XET activity ratio of *Equisetum* HTG is approximately 3500‐fold higher than that of known XTHs (Hrmova *et al*., 2007; Fry *et al*., 2008a; Maris *et al*., 2011), including those from *Equisetum*, marking it out as a unique enzyme.

We characterized native *Equisetum* HTG and the corresponding protein heterologously produced in the yeast *Pichia*. Studying the enzyme extracted from *Equisetum* provides reliable information on the natural plant protein, with correct post‐translational modifications, e.g. *N‐*glycosylation, while use of the *Pichia*‐produced protein ensured that no contaminating proteins acting on plant polysaccharides are present.

HTG has an extremely high affinity for its XGO acceptor substrates (*K*_M_ < 1 μm) and a high catalytic efficiency (Table 1). Its affinities for its soluble donor substrates (Figure 4e) are comparable to those of known XTHs. It is impossible to provide precise kinetic data (e.g. *K*_M_) for its insoluble donor substrate, cellulose; however, Figure 4(b) shows that, at a high cellulose concentration, HTG has a CXE activity that is comparable to its MXE activity.

Filter paper is a cellulosic donor substrate for HTG. Pre‐treatment of the paper with NaOH (increasing the purity of the cellulose and converting it to the cellulose II allomorph; Kroon‐Batenburg and Kroon, 1997) rendered it a better donor substrate for the *Pichia*‐produced enzyme (Figure 4b). This NaOH effect was less pronounced for the native *Equisetum* enzyme (Figure 1e; ‘PP’ versus ‘AP’). The presence of an inert protein, BSA, increased the ability of *Pichia*‐produced HTG to act on cellulose (Figure 4b), probably because BSA prevented irreversible binding of HTG to the paper and consequent loss of its ability to move to new cellulose sites where catalysis may continue. In support of this interpretation, the BSA effect was much less pronounced during the first few minutes of the reaction, and was negligible when soluble donor substrates were tested (Figure S6).

Modelling the 3D structure of HTG in comparison with two angiosperm XTHs suggested that evolution of *HTG* from a presumed ancestral *XTH* gene involved relatively few amino acid substitutions during the 370 million year history of the Equisetopsida. The key amino acid substitutions that are likely to account for HTG's lax donor substrate specificity are a replacement of a conserved Trp of conventional XTHs by Pro at position 10 in EfHTG, replacement of a conserved Gly by Ser at position 34, and replacement of a conserved Arg by Leu at position 245 (Figure S3b).

It is unknown whether, during equisetopsid evolution, the polysaccharide MLG pre‐dated the enzyme HTG. However, as the glucose residues of cellulose, but not MLG, occupy the same positions in the enzyme's active site as those of xyloglucan, it seems plausible that HTG targeted cellulose before adapting to MLG.

In *Equisetum*, both MLG and cellulose predominate in secondary walls, whereas HTG's acceptor substrate (xyloglucan) predominates in primary walls (Leroux *et al*., 2011). We suggest that the enzyme may inter‐connect the primary and secondary wall layers by forming cellulose–xyloglucan and MLG–xyloglucan linkages, an action that may play a unique strengthening role in the *Equisetum* stem. In agreement with this hypothesis, extractable MXE activity (and thus presumably the HTG protein and its associated CXE activity) is maximal in ageing *Equisetum* stems, and is much lower in young, fast‐growing shoots and in callus cultures (Fry *et al*., 2008a; Mohler *et al*., 2013). This pattern is the converse of that observed for XET activity (predominantly attributable to XTH proteins), which peaks in growing tissues, especially the intercalary meristem, the rapidly elongating and short‐lived *E. arvense* strobilus stem, and *E. fluviatile* callus (Mohler *et al*., 2013).

HTG's ability to form cellulose–xyloglucan and MLG–xyloglucan bonds potentially offers valuable new biotechnological opportunities to stably ‘functionalize’ these biomass polysaccharides without generating pollutant by‐products. The basis of such functionalization may include covalently bonding cellulose or MLG to xyloglucan oligosaccharides that have themselves previously been derivatized by loading with a valuable ‘cargo’ such as a dye. As an example, the HTG‐generated orange spots in Figure 1(e) are a paper–XGO–sulforhodamine covalent complex, the colour of which remains during very harsh treatments (6 m NaOH at 37°C, phenol/acetic acid at 70°C, and boiling SDS). We suggest that, in addition to dyes, cargoes may include disinfectants, tamper‐proof markings for legal documents, antibiotics, drugs, or laboratory reagents (for indicator papers).

Furthermore, introduction of *EfHTG* into angiosperms will potentially enable covalent MLG–xyloglucan bonding (in cereals) and cellulose–xyloglucan bonding (in any land plant), reactions that do not occur naturally in angiosperms. Such bonding may enhance the mechanical strength of crops, e.g. their resistance to damage by wind and heavy rain.

In conclusion, we have characterized HTG, the first predominantly *hetero*‐transglycanase from a plant and the first transglycanase shown to target insoluble cellulose, the major component of biomass. Although HTG appears to be confined to a single genus of land plants, *Equisetum*, potentially valuable biotechnological applications for crop plants and their constituent polysaccharides may be proposed.

## Experimental procedures

### Materials

*Equisetum fluviatile* (water horsetail), *E. arvense* (common horsetail) and *Holcus lanatus* (Yorkshire fog grass) were grown outdoors in Edinburgh, UK.

The xyloglucan oligosaccharide names are abbreviated in the usual way, e.g. G, Glc; Gol, glucitol; X, Xyl‐Glc; L, Gal‐Xyl‐Glc (Fry *et al*., 1993). Other oligosaccharide abbreviations include: Gal6‐ol, (1→4)‐β‐d‐galactohexaitol; GalA6–ol, reduced (1→4)‐α‐d‐galacturonohexaose; Malt6‐ol, (1→4)‐α–glucohexaitol; Man6–ol, (1→4)‐β‐d‐mannohexaitol; Xyl6–ol, (1→4)‐β‐d–xylohexaitol. MLG oligosaccharides are named as, for example, G4G4G3G, where G represents β‐d‐glucose and 3 and 4 represent (1→3) and (1→4) bonds respectively.

Lichenase (from *Bacillus subtilis*), xyloglucan‐inactive cellulase (from *Aspergillus niger*), barley MLG (‘β‐glucan’; medium viscosity), Iceland moss MLG (‘lichenan’) and most oligosaccharides were obtained from Megazyme Inc. ( http://www.megazyme.com/) Substrates prepared in‐house included water‐soluble cellulose acetate (Fry *et al*., 2008a), galacto‐oligosaccharides (Popper and Fry, 2008), galacturono‐oligosaccharides (García‐Romera and Fry, 1994) and xyloglucan oligosaccharides (Hetherington and Fry, 1993). MLG oligosaccharides (MLGOs) with non‐reducing terminal (1→3)‐ or (1→4)‐linkages were prepared by digestion of barley MLG with cellulase or lichenase, respectively, and purified on Bio–Gel P‐2 or P‐4 (Bio‐Rad Inc.; http://www.bio-rad.com/).

Most [^3^H]oligosaccharidyl alditols were prepared by reduction of the oligosaccharides with NaB^3^H_4_ (Hetherington and Fry, 1993), and purified by TLC or paper chromatography (Fry, 2000). [^3^H]GGXXXGol was prepared as described by Mohler *et al*. (2013). [^3^H]GXXGol was produced by digestion of [^3^H]XXXGol with α‐xylosidase (Figure S2 in Franková and Fry, 2012). The [^3^H]oligosaccharides obtained were typically of specific radioactivity 40–900 MBq μmol^−1^.

Merck silica gel 60 TLC plates were obtained from VWR ( https://uk.vwr.com/). Solvents were obtained from Fisher Scientific ( www.fisher.co.uk/). Most other chemicals were obtained from Sigma–Aldrich ( www.sigmaaldrich.com/united-kingdom.html).

### HTG purification from *Equisetum fluviatile*

Fresh late‐season *E. fluviatile* stems were ground with a pestle and mortar in 300 mm succinate, 10 mm CaCl_2_, 20 mm ascorbic acid, 15% v/v glycerol, containing 3% w/v polyvinylpolypyrrolidone, pH 5.5 (Na^+^), at 5°C. Solid (NH_4_)_2_SO_4_ was added slowly with stirring. In 10%‐saturated (NH_4_)_2_SO_4_ increments, precipitated proteins were pelleted by centrifugation (8000 ***g*** at 4°C for 15 min), re‐dissolved in 50 mm succinate (Na^+^), pH 5.5, and stored at −80°C. Protein contents were estimated by the Bradford micro‐assay (Bradford, 1976).

The 20%‐saturated (NH_4_)_2_SO_4_‐precipitated fraction was subjected to gel‐permeation chromatography on Bio‐Gel P‐100 [bed volume 87 cm^3^, equilibrated with 300 mm citrate (Na^+^), pH 6.3]. The column was calibrated using 5–40 MDa dextran, cytochrome *c*_551_ (Ambler, 1974; kindly provided by the late Professor R.P. Ambler; University of Edinburgh) and CoCl_2_.

Jack‐bean (*Canavalia ensiformis*) concanavalin A–Sepharose 4B beads (1‐ml bed) were washed for 1 h with 50 mm citrate (Na^+^, pH 6.3) containing CaCl_2_, MnCl_2_ and MgCl_2_ (1 mm each). Pooled MXE‐active fractions from Bio‐Gel P‐100 (Figure S1b) were supplemented with BSA (10 mg ml^−1^), applied to the column, and eluted in wash buffer containing 0–500 mm methyl α‐d‐mannopyranoside. MXE and XET activities in fractions were corrected for the slight inhibitory effect of each eluent.

A Rotofor IEF apparatus (Bio‐Rad) was used according to the manufacturer's instructions. Internal surfaces of the equipment were washed with 0.25% v/v Triton X‐100. Electrophoresis was performed at 10 W until the voltage and current stabilized. The pH of the fractions was immediately measured, and the fractions were stored at −80°C. For broad‐range IEF, shoot extracts of *E. fluviatile* gathered in May were dialysed against 10 mm succinate (Na^+^, pH 5.5), and mixed with Bio‐Lyte ampholytes (pH 3–10; Bio‐Rad Inc.). For narrow‐range IEF, shoot extracts of *E. fluviatile* gathered in September were precipitated with 10–20%‐saturated (NH_4_)_2_SO_4_, then electrophoresed in Servalyte ampholytes (pH 3–5; Serva; http://www.serva.de/) containing 0.05% Triton X‐100. In both cases, 20 μl of each fraction was assayed for MXE and XET activity, and 5 μl of selected fractions was assayed for CXE.

SDS–PAGE was performed as described by Laemmli (1970). The stacking and resolving gels were 4% and 12% respectively; electrophoresis was performed at 75 V for approximately 15 min, then 100 V for approximately 75 min.

Native gel electrophoresis was performed at 6°C with 4.3% acrylamide in 67 mm Tris (phosphate^−^, pH 6.7) as the stacking gel, 7.5% acrylamide in 376 mm Tris (Cl^−^, pH 8.9) as the resolving gel, 5 mm Tris and 38 mm glycine (pH 8.3) as the electrode buffer, with a current of 20 mA for 25 min then 40 mA for 3 h. Three lanes of the gel were excised, rinsed for 2 × 15 min in 0.3 m citrate (Na^+^, pH 6.3), placed on Whatman No. 1 dot‐blot test papers (see below), and incubated for 1 h. The papers were then washed in acidified ethanol until free of unreacted XXXG–sulforhodamine, and photographed under 254‐nm UV. A fourth lane was stained using Coomassie brilliant blue. To estimate HTG concentration, we silver‐stained SDS gels of two independent preparations of 31‐kDa *Equisetum* HTG alongside a dilution series of ovalbumin (30, 15, 7.5, 3.8, 1.9 and 0.94 ng per well), and quantified the bands using labworks 4.6 image analysis ( http://www.perkinelmer.co.uk/) and imagej ( http://imagej.nih.gov/ij/) Software.

### Mass spectrometry

The full details for mass spectrometry are given in Methods S1. Coomassie‐stained bands from SDS gels were digested with trypsin, and solubilized peptides were collected. A trypsin‐only blank allowed internal calibration. Aliquots of the digests were analysed by MALDI–TOF MS with an α‐cyano‐4‐hydroxycinnamic acid matrix on a Voyager DE‐STR MALDI–TOF MS (Applied Biosystems; www.appliedbiosystems.com/). For LC–MS analysis, the sample was de‐salted (Rappsilber *et al*., 2003) and passed through an Agilent ( www.agilent.co.uk/) 1200 Series HPLC with a PicoTip emitter (FS 360‐100‐8‐N‐20‐C12, New Objective; http://www.newobjective.com/). Processed spectra were searched against the National Center for Biotechnology Information non‐redundant database and an *E. fluviatile* transcriptome database via in‐house‐licensed mascot software.

### *Equisetum* transcriptome sequencing

Lateral shoot tissue (approximately 500 mg) of a single *E. fluviatile* individual was finely ground in liquid N_2_ and mixed with 3 ml TRIzol (Invitrogen; www.lifetechnologies.com/). The suspension was centrifuged at 12 000 ***g*** (4°C, 5 min), and extracted with 0.2 volumes of chloroform. The aqueous phase was mixed with 0.54 volumes of ethanol and RNA purified from it by use of a PureLink RNA Mini Kit (Life Technologies; www.lifetechnologies.com). cDNA was synthesized and normalized with Mint and Trimmer kits (Evrogen; www.evrogen.com/) and used for 454 sequencing (Roche; www.454.com) at Edinburgh Genomics ( https://genomics.ed.ac.uk). Raw data were assembled with Roche Newbler assembler version 2.5 (268 000 reads; length 249 ± 111 nucleotides; mean ± SD).

### Gene amplification and cloning

3′‐ and 5′‐RACE yielded full‐length *EfHTG* cDNA sequences; full details are provided in Methods S1. PCR products were ligated into pJET1.2 vector (Life Technologies) (map available from http://www.bioinfo.pte.hu/f2/pict_f2/pJETmap.pdf), which was used to transform *Escherichia coli* strain DH5α. Ampicillin‐resistant colonies were screened by PCR, and PCR products of appropriate sizes were used directly in Sanger DNA sequencing.

To express EfHTG as a fusion protein from the *Pichia* pPICZαA vector (Life Technologies), we amplified cDNA with Phusion (New England Biolabs; www.neb.com) polymerase and HTG‐specific primers, which introduced a 5′ *Eco*RI site immediately upstream of the sequence encoding the putative N‐terminus of mature EfHTG, and replaced the EfHTG stop codon with an *Xba*I site. PCR products were cloned in DH5α in the pJET1.2 vector, and a recombinant vector lacking PCR‐induced mutations was purified from a single colony. The EfHTG gene was then excised from the recombinant vector by complete digestion with *Xba*I and partial digestion with *Eco*RI (as EfHTG contains an internal *Eco*RI site), gel‐purified, ligated into pPICZαA, and cloned into *E. coli* strain DH5α.

### Recombinant EfHTG production in *Pichia pastoris*

Full details for heterologous protein production are given in Methods S1. *E. coli* carrying recombinant pPICZαA was selected on zeocin. Purified recombinant pPICZαA vector from a single colony was linearized by digestion with *Sac*I, and used to transform *Pichia pastoris* strain SMD1168H by electroporation, with selection on zeocin. Positive colonies were confirmed by PCR with EfHTG‐specific primers, and grown overnight in a liquid medium. Cells were recovered by centrifugation, and re‐suspended in expression medium (containing 1% methanol). After 16 h, cell‐free medium was assayed for transglucanase activities.

The recombinant EfHTG carried a C‐terminal His_6_ tag allowing purification by affinity chromatography on Ni^2+^‐charged chelating Sepharose (GE Healthcare; www3.gehealthcare.co.uk/). Two elution protocols were successfully used: one for testing substrate specificity (Figures 4g and 6c) and one for Western blotting (Figure 3) (see Methods S1).

### Modelling the three‐dimensional structure of HTG

We used the iTASSER server (Zhang, 2008) to create an initial HTG homology model. This was then superposed onto the structures for PttXET (Johansson *et al*., 2004) and TmNXG1 (Mark *et al*., 2009) (PDB IDs 1UMZ and 2VH9, respectively). We created the complete XLLGXLG (pentadecasaccharide) substrate molecule by merging the XLLG ligand present in the TmNXG1 crystal structure with the XLG ligand present in the PttXET structure, manually adjusting the sugar residue in the −1 position to a 1S3 skew‐boat conformation and creating a β‐(1→4) covalent bond. We energy‐minimized this molecule using maestro version 9.3 (Schrödinger; www.schrodinger.com) to clear steric clashes, then removed the three β‐galactose residues to create XXXGXXG. We added this dodecasaccharide molecule to the HTG model, and manually adjusted the positions of HTG side chains observed to interact with the ligand to match their positions in the appropriate ligand‐bound structure, by first selecting the nearest rotamer present in the PyMOL rotamer library using the pymol molecular graphics system version 1.6.0.0 (Schrödinger), then making fine adjustments by hand. We then maximized favourable protein–ligand interactions by energy‐minimizing this complex. Complexes of PttXET and TmNXG1 with XXXGXXG were created in a similar manner for comparison.

### Thin‐layer chromatography

TLC was performed on Merck silica gel plates in butan‐1‐ol/acetic acid/water (2:1:1). A malto‐oligosaccharide ladder plus glucose was used as the marker mixture. Sugars were stained with thymol/H_2_SO_4_ (Jork *et al*., 1994); radioactive spots were detected fluorographically.

### Radiochemical transglucanase assays

Reaction mixtures (Fry *et al*., 1992, 2008a) with soluble donor substrates (e.g. xyloglucan or MLG) typically contained non‐radioactive soluble polysaccharide (1–10 mg ml^−1^) or cellohexaose (7.6–76 mm), 1–2 kBq [^3^H]XXXGol (100–900 MBq μmol^−1^), an enzyme source, 0.2% chlorobutanol (an antimicrobial agent) and buffer [25–200 mm succinate (Na^+^), pH 5.5, or citrate (Na^+^), pH 6.3], final volume 20–150 μl, and were incubated for 1–60 h at 20°C. The reaction was stopped with 20 μl formic acid.

When MLG or xyloglucan was used as the donor substrate, ^3^H‐labelled products were dried onto 4 × 6 cm of Whatman ( http://www.fisher.co.uk/1/3/whatman-qualitative-filter-paper) 3MM paper, which was then washed for 1–2 days in running tap‐water and dried; ^3^H‐labelled polymers that remained bound were assayed by scintillation counting in GoldStar ‘O’ scintillation fluid (Meridian; www.meridian-biotech.com/). Counting efficiencies of XET, MXE and CXE products were 24.6, 9.0 and 6.9% respectively, as determined in representative samples after acid hydrolysis and scintillation counting in water‐miscible scintillant (ScintiSafe 3; Fisher Scientific) with quench correction. The differences in efficiency indicate that the XET product was confined to the surface of the paper fibres, whereas the products of the other two activities were more intimately integrated within the fibres.

To determine the *K*_M_ of HTG for XXXGol, we mixed [^3^H]XXXGol with non‐radioactive XXXGol to various final specific radioactivities and thus various final concentrations. To determine *k*_cat_, we assayed IEF‐purified native *Equisetum* HTG at near‐optimal substrate concentrations (20 μm XXXGol, 7.4 mg ml^−1^ MLG).

When cellohexaose was the potential donor substrate, ^3^H‐labelled products were sought by TLC followed by fluorography. When water‐soluble cellulose acetate was the potential donor, the reaction was stopped with 1 m NaOH, which removes acetyl ester groups; the resulting cellulose was washed by repeated resuspension in water until the supernatant was no longer radioactive, and assayed for bound ^3^H.

For CXE assays, where insoluble cellulose was the donor, 20 μl of a solution containing the soluble components (^3^H‐labelled acceptor substrate, enzyme, buffer, chlorobutanol and, in some experiments, 1.1 mg ml^−1^ BSA) was pipetted onto 24–25 mg of dry Whatman No. 1 paper, and the moist paper was incubated in a tightly closed vial. In some experiments, the paper had been pre‐treated for 16 h at 20°C in 6 m NaOH, rinsed in water and dried (weight loss approximately 1.2%). The enzymic reaction was stopped with 20 μl formic acid; the paper was washed for 1–2 d in water, dried and assayed for bound ^3^H.

In the case of ^3^H‐labelled acceptor substrates that themselves hydrogen‐bond to cellulose (cello‐, xylo‐, manno‐ and large MLG‐oligosaccharides), and thus fail to completely wash off paper, transglucanase products were not dried onto paper. Instead the reaction was stopped with 500 μl of 8% formic acid (containing non‐radioactive carrier polysaccharides: 0.7 mg ml^−1^ blue dextran, 0.35 mg ml^−1^ barley MLG and 0.35 mg ml^−1^ tamarind (*Tamarindus indica*) xyloglucan), and the mixture was then made up to 70% ethanol. After at least 16 h storage, the polysaccharides were pelleted by centrifugation (4500 ***g*** for 10 min), re‐dissolved with gentle warming in 3 ml of 1% aqueous ammonium formate, and re‐precipitated with ethanol. Dissolution and precipitation were repeated twice more, and the final pellet was dissolved in 2 ml water and assayed for ^3^H in ScintiSafe 3.

### Cellulase digestion of radiolabelled CXE product

A 31‐mg sample of CXE product (formed by *Pichia*‐produced *Equisetum* HTG acting on filter paper pre‐treated with alkali) was thoroughly washed in 6 m NaOH (containing 1% NaBH_4_) at 20°C for 48 h, then washed with water until neutral, dried and incubated at 20°C in 500 μl buffer (pyridine/acetic acid/water; 1:1:98 v/v/v, pH 4.7) containing 0.5% chlorobutanol, with or without 0.5 units of xyloglucan‐inactive cellulase. A 28‐mg control sample of CXE product was incubated with buffer/chlorobutanol alone. After 72 h, 2.5 units of cellulase were added to each sample, and incubation continued. Solubilized radioactivity was assayed at intervals by scintillation counting at 33% efficiency. A portion of the 95‐h digest was analysed by TLC (three ascents), and strips were assayed for ^3^H by scintillation counting. A further portion of the same digest was run on the same TLC plate and stained with thymol/H_2_SO_4_. A third portion was analysed by paper chromatography on Whatman No. 1 paper in butan‐1‐ol/pyridine/water (4:3:4; with 105 h development by the descending method). Strips of the paper (1 or 2 cm) were assayed for ^3^H by scintillation counting.

### Dot‐blot transglucanase assays

Visual assays for transglucanase activities were based on the dot‐blot method (Fry, 1997). Paper (Whatman No. 1) was bathed in 1% w/v tamarind xyloglucan (‘XP’), 2% barley MLG (‘MP’), water (‘PP’) or alkali (6 m NaOH, ‘AP’) with gentle rocking for 1–8 h. AP sheets irreversibly shrank to 61% of their original area, but have an increased fibre surface area (Kalia *et al*., 2011). The papers were then washed thoroughly in water until neutral, dried, cut to 7 × 7 cm, quickly dipped in 4.8 μm XXXG–sulforhodamine (Miller *et al*., 2007) in 50% acetone and re‐dried; fluorescent acceptor substrate was thereby impregnated into the paper at approximately 1.3 μmol m^−2^. Aliquots (4.5 μl, or 6.5 μl in the case of AP) from a dilution series of enzymes precipitated from an extract of grass (*H. lanatus*) or horsetail (*E. fluviatile* or *E. arvense*) with 20%‐saturated (NH_4_)_2_SO_4_ were pipetted onto the papers with 1‐cm centre‐to‐centre spacing; buffer‐only controls were also included. After 21 h incubation at 20°C under humid conditions, unreacted XXXG–sulforhodamine was washed out with ethanol/formic acid/water (3:2:2 v/v/v), and insoluble fluorescent products of polysaccharide‐to‐oligosaccharide transglycosylation were recorded under 254‐nm UV excitation. In principle, PP and AP reveal only CXE activity, XP reveals XET + CXE activity, and MP reveals MXE + CXE activity. However, the grass extract contained traces of xyloglucan, and thus yielded a faint fluorescence on papers other than XP. The same papers were sequentially washed in 6 m NaOH (16 h at 20°C, solubilizing xyloglucan and MLG), water, 4% SDS (10 min at 98°C, then 16 h at 20°C), water, phenol/acetic acid/water (2:1:1 w/v/v; 16 h at 20°C), water and acetone. The papers were then incubated with lichenase [1 U ml^−1^ in pyridine/acetic acid/water (1:1:98 v/v/v, pH 4.7) containing 0.5% chlorobutanol] for 16 h at 20°C, and finally with cellulase (1 U ml^−1^, same conditions). After each step, papers were re‐photographed, always with identical UV exposure, camera settings and brightness/contrast adjustment. As expected, lichenase had no effect, as any MLG had been removed by NaOH. XP, MP and PP test papers shrank in NaOH; images are adjusted to uniform size. The difference in fluorescence between papers washed in ethanol/formic acid/water or NaOH indicates XET or MXE reaction products; residual fluorescence after NaOH treatment indicates CXE product (cellulose–XXXG–sulforhodamine conjugate).

## Supporting information


**Figure S1.** Fractionation of native *Equisetum* HTG.

**Figure S2.** Mass spectra of tryptic peptides of *E. fluviatile* HTG.

**Figure S3.** Deduced sequence of *E. fluviatile* HTG, compared with several other family GH16b proteins.

**Figure S4.** Rooted cladogram showing the relationship of HTG to other GH16b sub‐family members.

**Figure S5.** Inability of *Pichia*‐produced HTG to utilize cellohexaose as donor substrate.

**Figure S6.** Effect of BSA on the activity of *Pichia*‐produced HTG with soluble and insoluble donor substrates.

**Table S1.** Radiochemical characterization of cellulose–[^3^H]XXXGol generated by HTG extracted from *E. fluviatile* stems.

**Table S2.** A four‐step strategy for purification of native *Equisetum* HTG.

**Table S3.** Modelled interactions between xyloglucan and the active site in *Populus* XTH (PttXET) (Johansson *et al*., 2004), *Tropaeolum* XTH (TmNXG1) (Mark *et al*., 2009) and *Equisetum* HTG (present work).

**Methods S1.** Mass spectrometry, gene amplification, cloning, and heterologous protein production.
