# Supplementary material for: Hetero‐trans‐β‐glucanase, an enzyme unique to Equisetum plants, functionalizes cellulose
Source: Plant J. 2015 Aug 25;83(5):753–69. doi: 10.1111/tpj.12935 (PMC4950035; doi:10.1111/tpj.12935)
Supplement: Supplementary file 1 — Figure S1. Fractionation of native Equisetum HTG. [file TPJ-83-753-s001.pptx]

## Slide 1
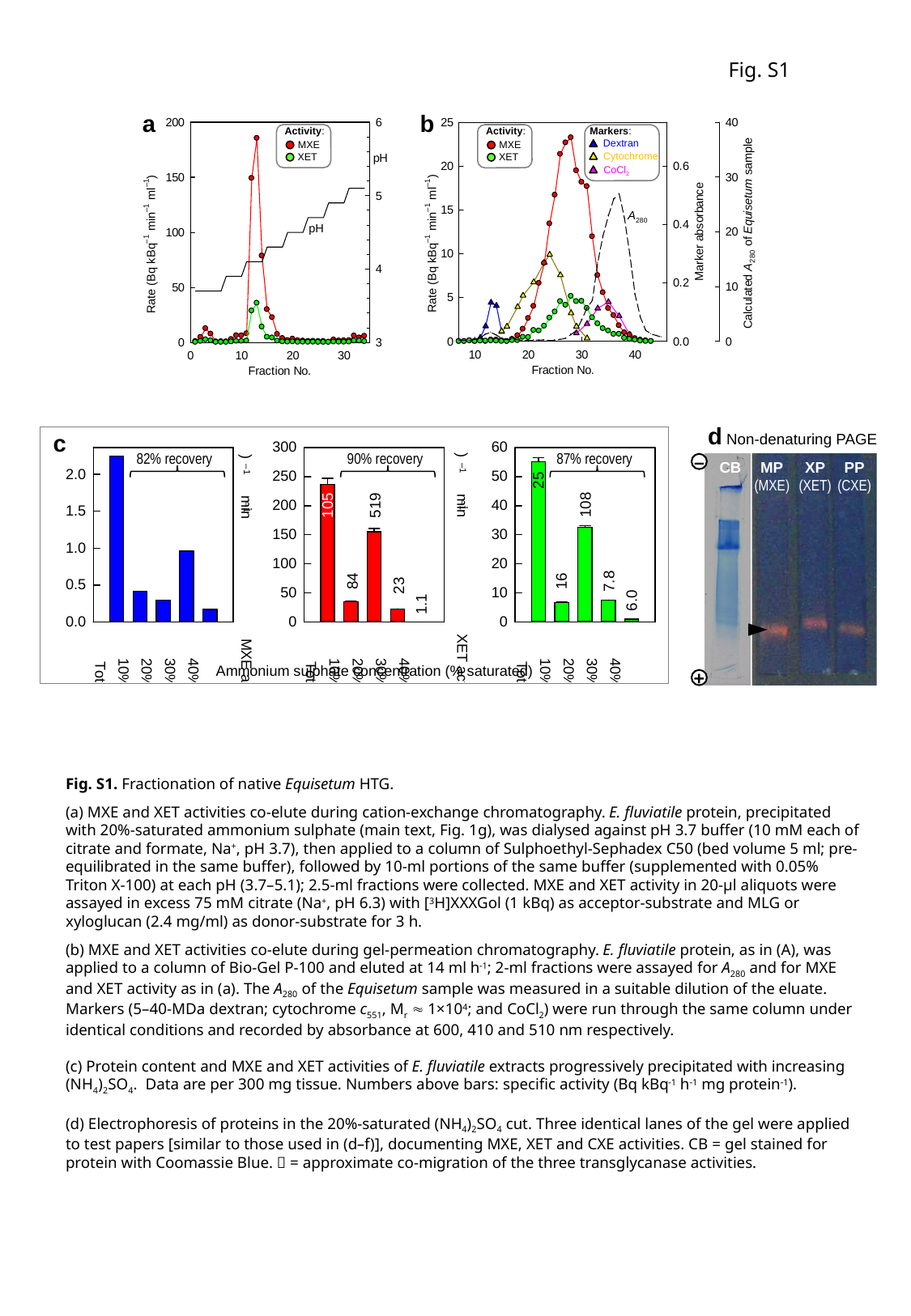

Fig. S1
a
b
Activity:
MXE
XET
Activity:
MXE
XET
Markers:
Dextran
Cytochrome
CoCl2
A280
pH
d Non-denaturing PAGE
MP
(MXE)
XP
(XET)
PP
(CXE)
CB
c
82% recovery
90% recovery
87% recovery
–
25
108
105
519
84
16
7.8
23
6.0
1.1
+
Fig. S1. Fractionation of native Equisetum HTG.
(a) MXE and XET activities co-elute during cation-exchange chromatography. E. fluviatile protein, precipitated with 20%-saturated ammonium sulphate (main text, Fig. 1g), was dialysed against pH 3.7 buffer (10 mM each of citrate and formate, Na+, pH 3.7), then applied to a column of Sulphoethyl-Sephadex C50 (bed volume 5 ml; pre-equilibrated in the same buffer), followed by 10-ml portions of the same buffer (supplemented with 0.05% Triton X-100) at each pH (3.7–5.1); 2.5-ml fractions were collected. MXE and XET activity in 20-µl aliquots were assayed in excess 75 mM citrate (Na+, pH 6.3) with [3H]XXXGol (1 kBq) as acceptor-substrate and MLG or xyloglucan (2.4 mg/ml) as donor-substrate for 3 h.
(b) MXE and XET activities co-elute during gel-permeation chromatography. E. fluviatile protein, as in (A), was applied to a column of Bio-Gel P-100 and eluted at 14 ml h−1; 2-ml fractions were assayed for A280 and for MXE and XET activity as in (a). The A280 of the Equisetum sample was measured in a suitable dilution of the eluate. Markers (5–40-MDa dextran; cytochrome c551, Mr  1×10^4^; and CoCl2) were run through the same column under identical conditions and recorded by absorbance at 600, 410 and 510 nm respectively.
(c) Protein content and MXE and XET activities of E. fluviatile extracts progressively precipitated with increasing (NH4)2SO4. Data are per 300 mg tissue. Numbers above bars: specific activity (Bq kBq−1 h−1 mg protein−1).
(d) Electrophoresis of proteins in the 20%-saturated (NH4)2SO4 cut. Three identical lanes of the gel were applied to test papers [similar to those used in (d–f)], documenting MXE, XET and CXE activities. CB = gel stained for protein with Coomassie Blue.  = approximate co-migration of the three transglycanase activities.
