# Supplementary material for: Hetero‐trans‐β‐glucanase, an enzyme unique to Equisetum plants, functionalizes cellulose
Source: Plant J. 2015 Aug 25;83(5):753–69. doi: 10.1111/tpj.12935 (PMC4950035; doi:10.1111/tpj.12935)
Supplement: Supplementary file 2 — Figure S2. Mass spectra of tryptic peptides of E. fluviatile HTG. [file TPJ-83-753-s002.pptx]

## Slide 1
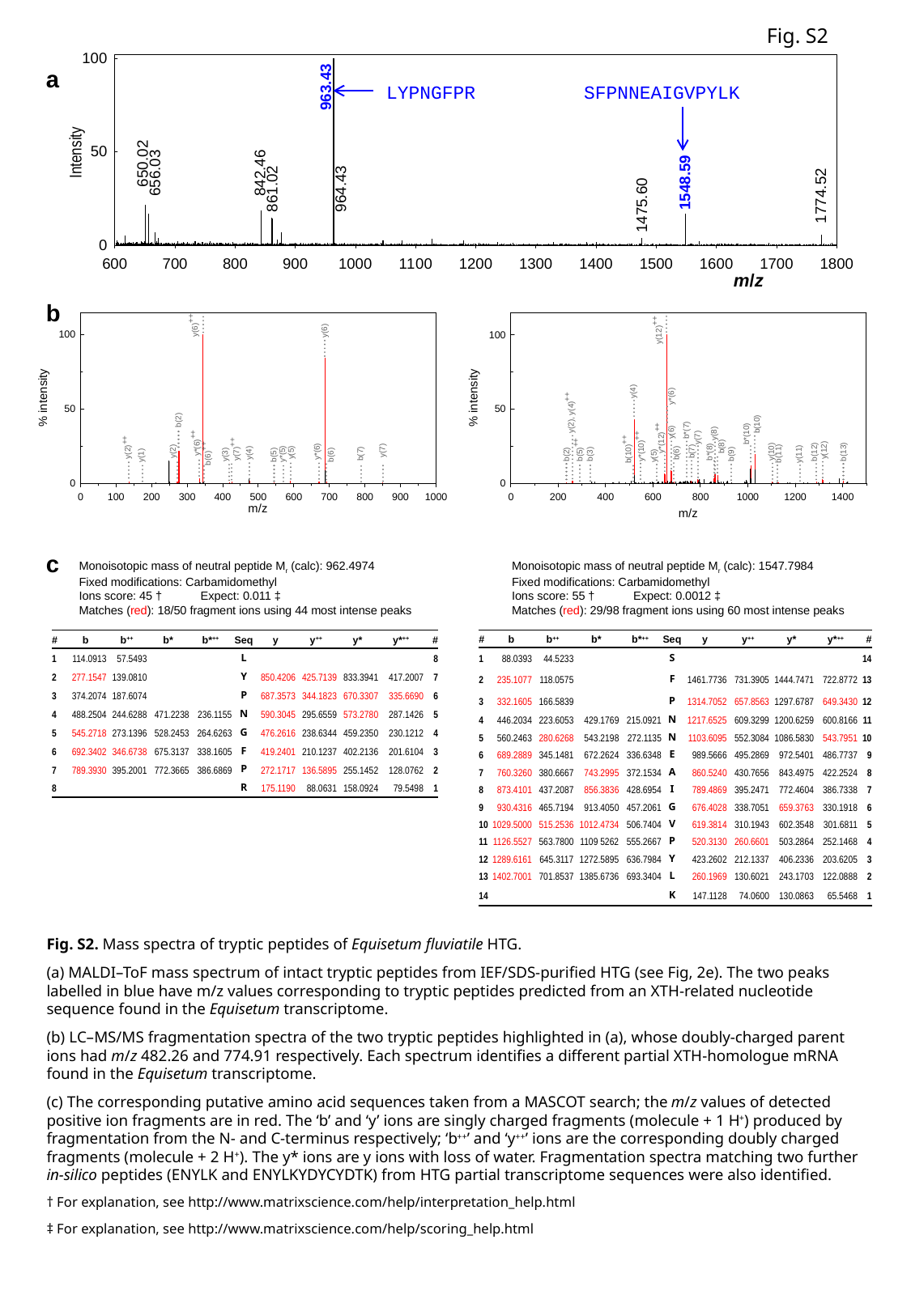

Fig. S2
LYPNGFPR
SFPNNEAIGVPYLK
963.43
650.02
656.03
842.46
1548.59
861.02
964.43
1774.52
1475.60
a
Intensity
b
c
Monoisotopic mass of neutral peptide Mr (calc): 962.4974
Fixed modifications: Carbamidomethyl
Ions score: 45 † 	Expect: 0.011 ‡
Matches (red): 18/50 fragment ions using 44 most intense peaks
Monoisotopic mass of neutral peptide Mr (calc): 1547.7984
Fixed modifications: Carbamidomethyl
Ions score: 55 † 	Expect: 0.0012 ‡
Matches (red): 29/98 fragment ions using 60 most intense peaks
| # | b | b++ | b\* | b\*++ | Seq | y | y++ | y\* | y\*++ | # |
| --- | --- | --- | --- | --- | --- | --- | --- | --- | --- | --- |
| 1 | 114.0913 | 57.5493 | | | L | | | | | 8 |
| 2 | 277.1547 | 139.0810 | | | Y | 850.4206 | 425.7139 | 833.3941 | 417.2007 | 7 |
| 3 | 374.2074 | 187.6074 | | | P | 687.3573 | 344.1823 | 670.3307 | 335.6690 | 6 |
| 4 | 488.2504 | 244.6288 | 471.2238 | 236.1155 | N | 590.3045 | 295.6559 | 573.2780 | 287.1426 | 5 |
| 5 | 545.2718 | 273.1396 | 528.2453 | 264.6263 | G | 476.2616 | 238.6344 | 459.2350 | 230.1212 | 4 |
| 6 | 692.3402 | 346.6738 | 675.3137 | 338.1605 | F | 419.2401 | 210.1237 | 402.2136 | 201.6104 | 3 |
| 7 | 789.3930 | 395.2001 | 772.3665 | 386.6869 | P | 272.1717 | 136.5895 | 255.1452 | 128.0762 | 2 |
| 8 | | | | | R | 175.1190 | 88.0631 | 158.0924 | 79.5498 | 1 |
| # | b | b++ | b\* | b\*++ | Seq | y | y++ | y\* | y\*++ | # |
| --- | --- | --- | --- | --- | --- | --- | --- | --- | --- | --- |
| 1 | 88.0393 | 44.5233 | | | S | | | | | 14 |
| 2 | 235.1077 | 118.0575 | | | F | 1461.7736 | 731.3905 | 1444.7471 | 722.8772 | 13 |
| 3 | 332.1605 | 166.5839 | | | P | 1314.7052 | 657.8563 | 1297.6787 | 649.3430 | 12 |
| 4 | 446.2034 | 223.6053 | 429.1769 | 215.0921 | N | 1217.6525 | 609.3299 | 1200.6259 | 600.8166 | 11 |
| 5 | 560.2463 | 280.6268 | 543.2198 | 272.1135 | N | 1103.6095 | 552.3084 | 1086.5830 | 543.7951 | 10 |
| 6 | 689.2889 | 345.1481 | 672.2624 | 336.6348 | E | 989.5666 | 495.2869 | 972.5401 | 486.7737 | 9 |
| 7 | 760.3260 | 380.6667 | 743.2995 | 372.1534 | A | 860.5240 | 430.7656 | 843.4975 | 422.2524 | 8 |
| 8 | 873.4101 | 437.2087 | 856.3836 | 428.6954 | I | 789.4869 | 395.2471 | 772.4604 | 386.7338 | 7 |
| 9 | 930.4316 | 465.7194 | 913.4050 | 457.2061 | G | 676.4028 | 338.7051 | 659.3763 | 330.1918 | 6 |
| 10 | 1029.5000 | 515.2536 | 1012.4734 | 506.7404 | V | 619.3814 | 310.1943 | 602.3548 | 301.6811 | 5 |
| 11 | 1126.5527 | 563.7800 | 1109 5262 | 555.2667 | P | 520.3130 | 260.6601 | 503.2864 | 252.1468 | 4 |
| 12 | 1289.6161 | 645.3117 | 1272.5895 | 636.7984 | Y | 423.2602 | 212.1337 | 406.2336 | 203.6205 | 3 |
| 13 | 1402.7001 | 701.8537 | 1385.6736 | 693.3404 | L | 260.1969 | 130.6021 | 243.1703 | 122.0888 | 2 |
| 14 | | | | | K | 147.1128 | 74.0600 | 130.0863 | 65.5468 | 1 |
Fig. S2. Mass spectra of tryptic peptides of Equisetum fluviatile HTG.
(a) MALDI–ToF mass spectrum of intact tryptic peptides from IEF/SDS-purified HTG (see Fig, 2e). The two peaks labelled in blue have m/z values corresponding to tryptic peptides predicted from an XTH-related nucleotide sequence found in the Equisetum transcriptome.
(b) LC–MS/MS fragmentation spectra of the two tryptic peptides highlighted in (a), whose doubly-charged parent ions had m/z 482.26 and 774.91 respectively. Each spectrum identifies a different partial XTH-homologue mRNA found in the Equisetum transcriptome.
(c) The corresponding putative amino acid sequences taken from a MASCOT search; the m/z values of detected positive ion fragments are in red. The ‘b’ and ‘y’ ions are singly charged fragments (molecule + 1 H+) produced by fragmentation from the N- and C-terminus respectively; ‘b++’ and ‘y++’ ions are the corresponding doubly charged fragments (molecule + 2 H+). The y* ions are y ions with loss of water. Fragmentation spectra matching two further in-silico peptides (ENYLK and ENYLKYDYCYDTK) from HTG partial transcriptome sequences were also identified.
† For explanation, see http://www.matrixscience.com/help/interpretation_help.html
‡ For explanation, see http://www.matrixscience.com/help/scoring_help.html
