# Supplementary material for: Hetero‐trans‐β‐glucanase, an enzyme unique to Equisetum plants, functionalizes cellulose
Source: Plant J. 2015 Aug 25;83(5):753–69. doi: 10.1111/tpj.12935 (PMC4950035; doi:10.1111/tpj.12935)
Supplement: Supplementary file 3 — Figure S3. Deduced sequence of E. fluviatile HTG, compared with several other family GH16b proteins. [file TPJ-83-753-s003.pptx]

## Slide 1
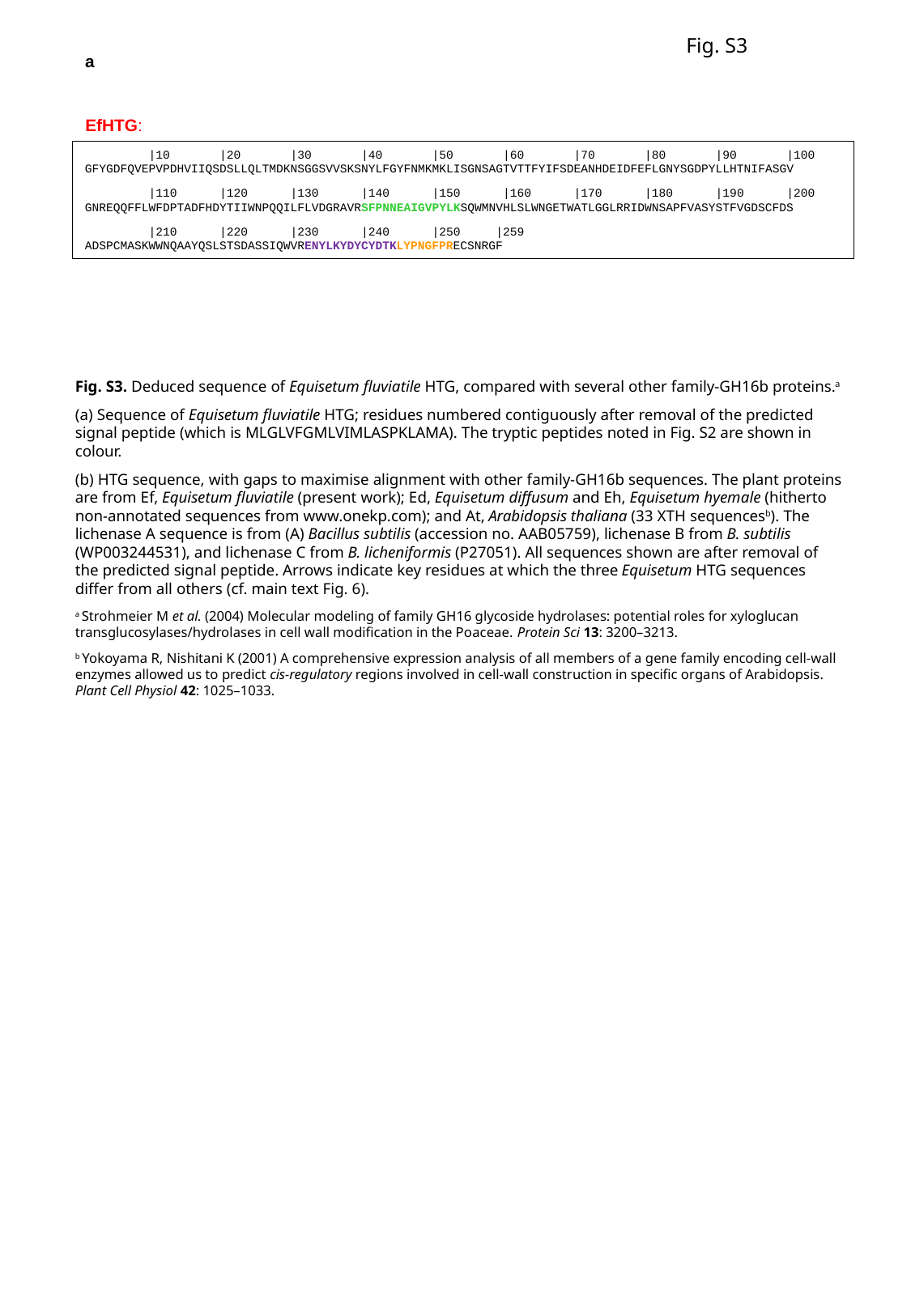

Fig. S3
a
EfHTG:
 |10 |20 |30 |40 |50 |60 |70 |80 |90 |100
GFYGDFQVEPVPDHVIIQSDSLLQLTMDKNSGGSVVSKSNYLFGYFNMKMKLISGNSAGTVTTFYIFSDEANHDEIDFEFLGNYSGDPYLLHTNIFASGV
 |110 |120 |130 |140 |150 |160 |170 |180 |190 |200
GNREQQFFLWFDPTADFHDYTIIWNPQQILFLVDGRAVRSFPNNEAIGVPYLKSQWMNVHLSLWNGETWATLGGLRRIDWNSAPFVASYSTFVGDSCFDS
 |210 |220 |230 |240 |250 |259
ADSPCMASKWWNQAAYQSLSTSDASSIQWVRENYLKYDYCYDTKLYPNGFPRECSNRGF
Fig. S3. Deduced sequence of Equisetum fluviatile HTG, compared with several other family-GH16b proteins.a
(a) Sequence of Equisetum fluviatile HTG; residues numbered contiguously after removal of the predicted signal peptide (which is MLGLVFGMLVIMLASPKLAMA). The tryptic peptides noted in Fig. S2 are shown in colour.
(b) HTG sequence, with gaps to maximise alignment with other family-GH16b sequences. The plant proteins are from Ef, Equisetum fluviatile (present work); Ed, Equisetum diffusum and Eh, Equisetum hyemale (hitherto non-annotated sequences from www.onekp.com); and At, Arabidopsis thaliana (33 XTH sequencesb). The lichenase A sequence is from (A) Bacillus subtilis (accession no. AAB05759), lichenase B from B. subtilis (WP003244531), and lichenase C from B. licheniformis (P27051). All sequences shown are after removal of the predicted signal peptide. Arrows indicate key residues at which the three Equisetum HTG sequences differ from all others (cf. main text Fig. 6).
a Strohmeier M et al. (2004) Molecular modeling of family GH16 glycoside hydrolases: potential roles for xyloglucan transglucosylases/hydrolases in cell wall modification in the Poaceae. Protein Sci 13: 3200–3213.
b Yokoyama R, Nishitani K (2001) A comprehensive expression analysis of all members of a gene family encoding cell-wall enzymes allowed us to predict cis-regulatory regions involved in cell-wall construction in specific organs of Arabidopsis. Plant Cell Physiol 42: 1025–1033.

## Slide 2
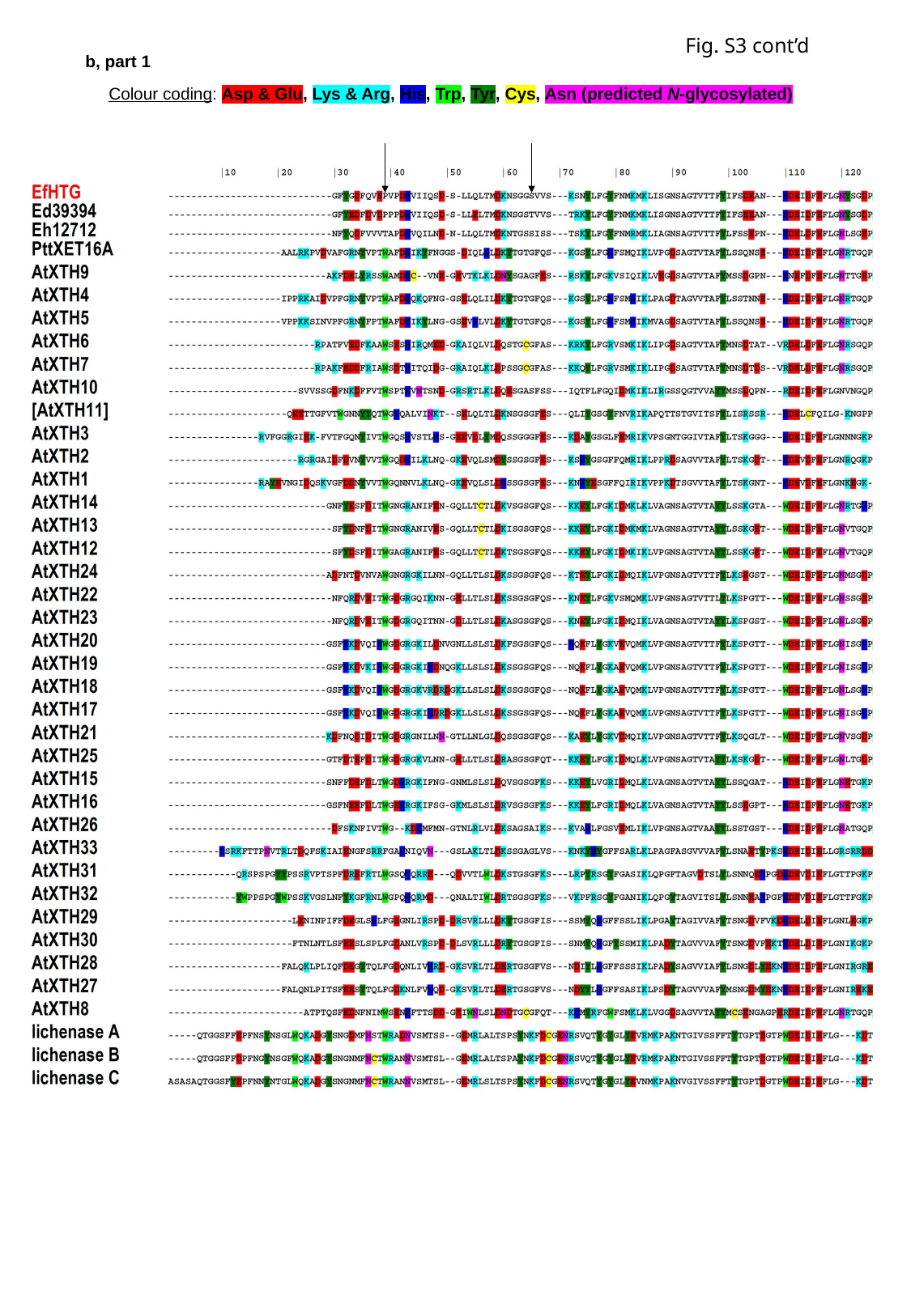

Fig. S3 cont’d
b, part 1
Colour coding: Asp & Glu, Lys & Arg, His, Trp, Tyr, Cys, Asn (predicted N-glycosylated)

## Slide 3
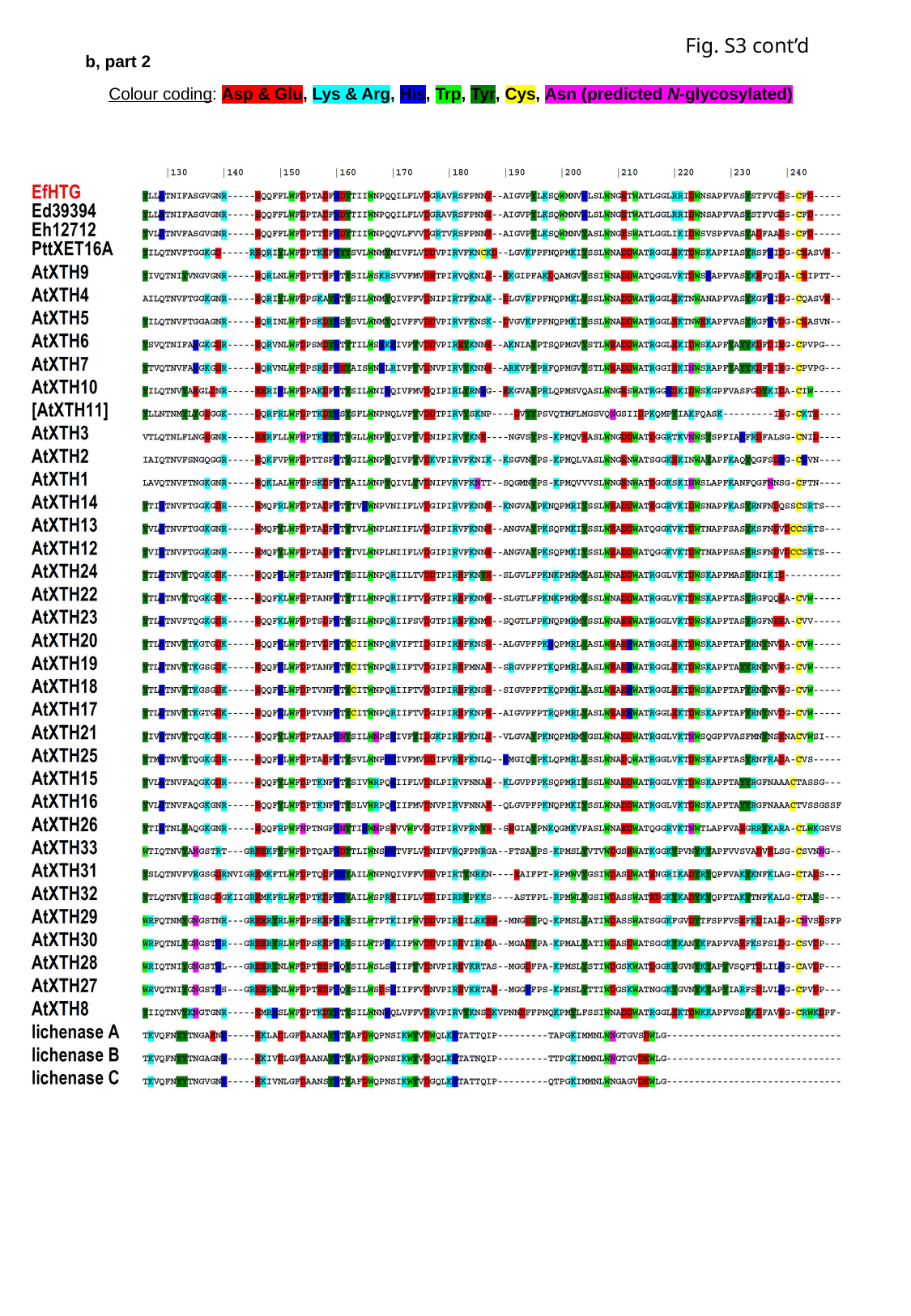

Fig. S3 cont’d
b, part 2
Colour coding: Asp & Glu, Lys & Arg, His, Trp, Tyr, Cys, Asn (predicted N-glycosylated)

## Slide 4
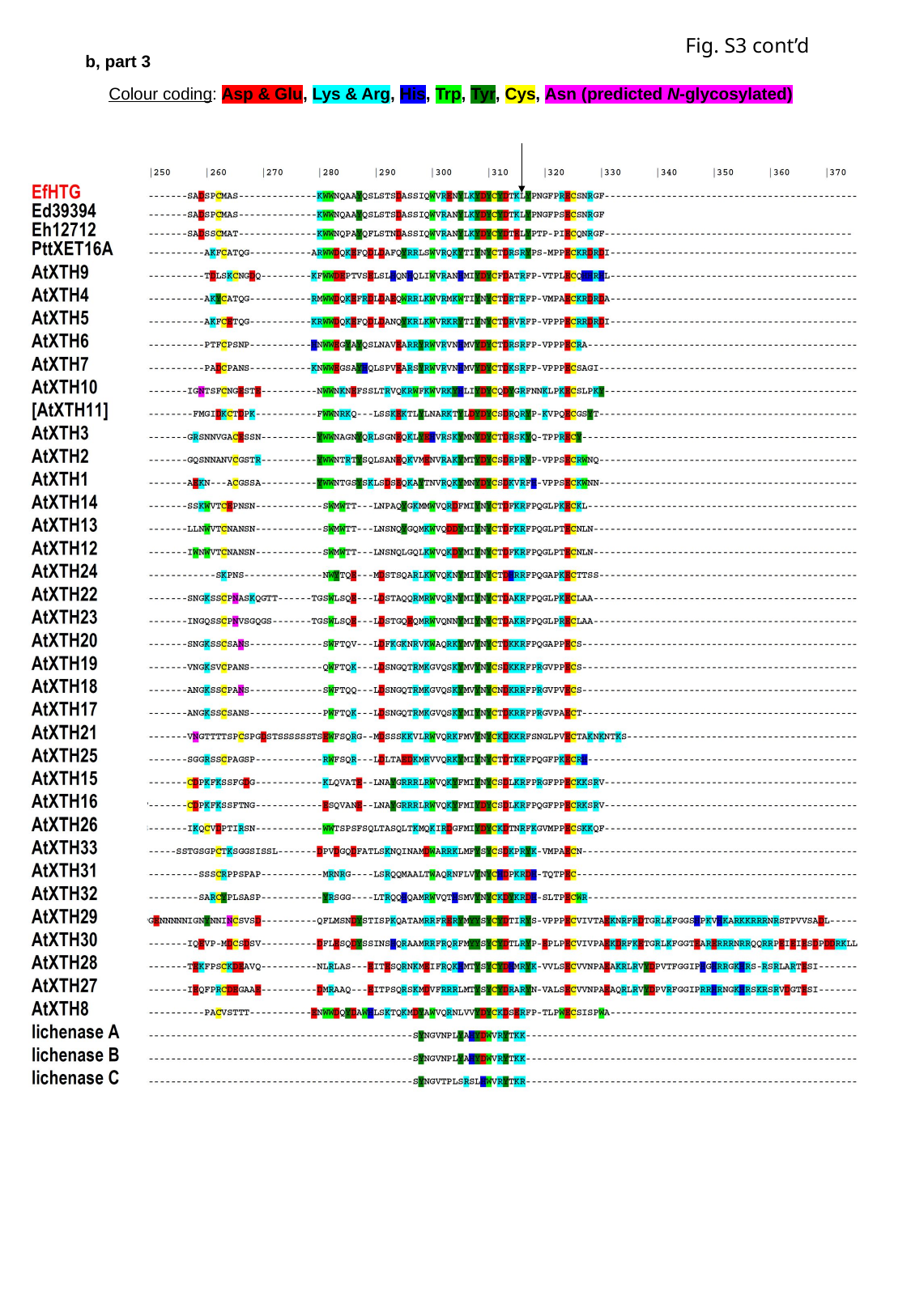

Fig. S3 cont’d
b, part 3
Colour coding: Asp & Glu, Lys & Arg, His, Trp, Tyr, Cys, Asn (predicted N-glycosylated)
