# Supplementary material for: Hetero‐trans‐β‐glucanase, an enzyme unique to Equisetum plants, functionalizes cellulose
Source: Plant J. 2015 Aug 25;83(5):753–69. doi: 10.1111/tpj.12935 (PMC4950035; doi:10.1111/tpj.12935)
Supplement: Supplementary file 4 — Figure S4. Rooted cladogram showing the relationship of HTG to other GH16b sub‐family members. [file TPJ-83-753-s004.pptx]

## Slide 1
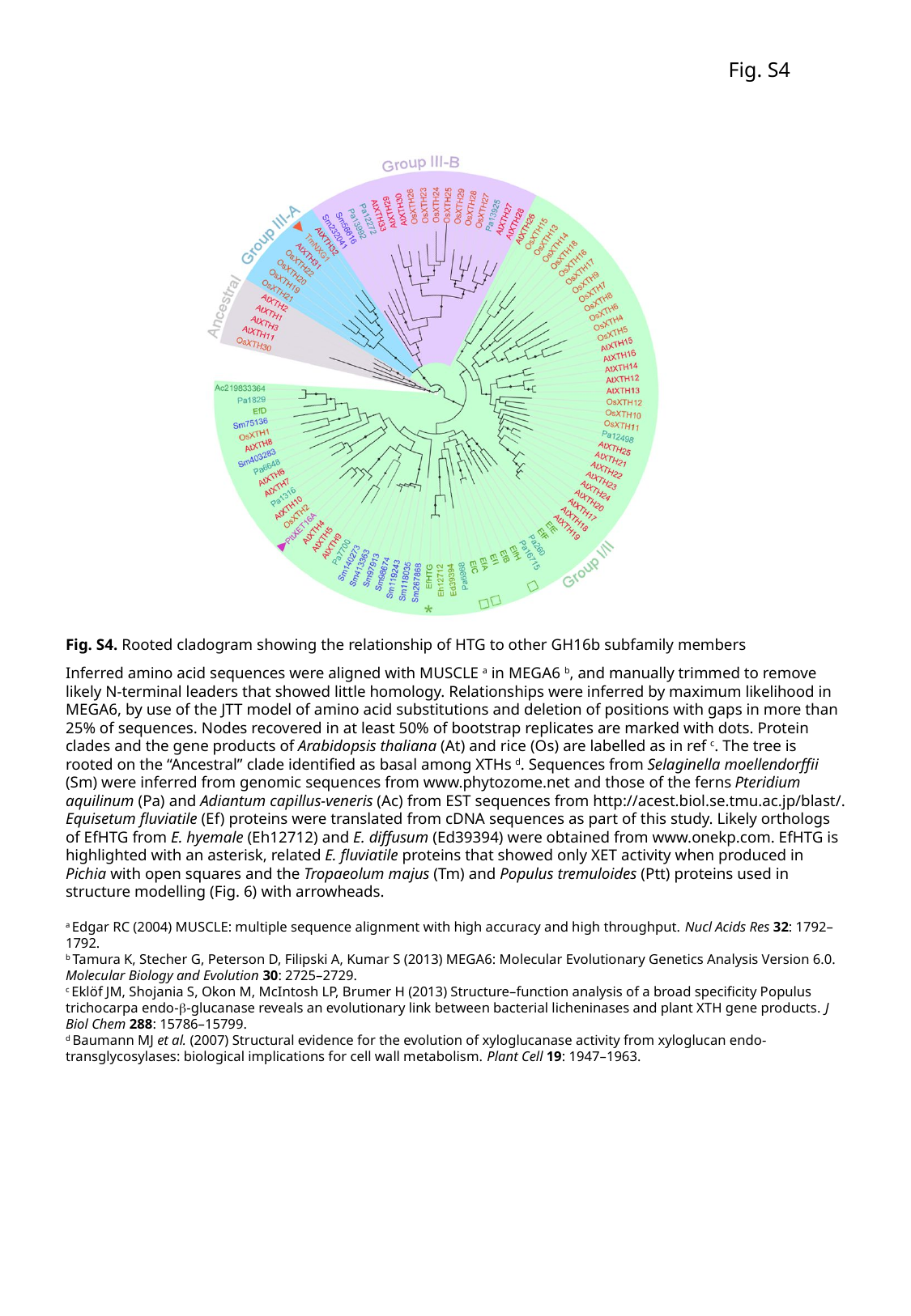

Fig. S4
Fig. S4. Rooted cladogram showing the relationship of HTG to other GH16b subfamily members
Inferred amino acid sequences were aligned with MUSCLE a in MEGA6 b, and manually trimmed to remove likely N-terminal leaders that showed little homology. Relationships were inferred by maximum likelihood in MEGA6, by use of the JTT model of amino acid substitutions and deletion of positions with gaps in more than 25% of sequences. Nodes recovered in at least 50% of bootstrap replicates are marked with dots. Protein clades and the gene products of Arabidopsis thaliana (At) and rice (Os) are labelled as in ref c. The tree is rooted on the “Ancestral” clade identified as basal among XTHs d. Sequences from Selaginella moellendorffii (Sm) were inferred from genomic sequences from www.phytozome.net and those of the ferns Pteridium aquilinum (Pa) and Adiantum capillus-veneris (Ac) from EST sequences from http://acest.biol.se.tmu.ac.jp/blast/. Equisetum fluviatile (Ef) proteins were translated from cDNA sequences as part of this study. Likely orthologs of EfHTG from E. hyemale (Eh12712) and E. diffusum (Ed39394) were obtained from www.onekp.com. EfHTG is highlighted with an asterisk, related E. fluviatile proteins that showed only XET activity when produced in Pichia with open squares and the Tropaeolum majus (Tm) and Populus tremuloides (Ptt) proteins used in structure modelling (Fig. 6) with arrowheads.
a Edgar RC (2004) MUSCLE: multiple sequence alignment with high accuracy and high throughput. Nucl Acids Res 32: 1792–1792.
b Tamura K, Stecher G, Peterson D, Filipski A, Kumar S (2013) MEGA6: Molecular Evolutionary Genetics Analysis Version 6.0. Molecular Biology and Evolution 30: 2725–2729.
c Eklöf JM, Shojania S, Okon M, McIntosh LP, Brumer H (2013) Structure–function analysis of a broad specificity Populus trichocarpa endo-β-glucanase reveals an evolutionary link between bacterial licheninases and plant XTH gene products. J Biol Chem 288: 15786–15799.
d Baumann MJ et al. (2007) Structural evidence for the evolution of xyloglucanase activity from xyloglucan endo-transglycosylases: biological implications for cell wall metabolism. Plant Cell 19: 1947–1963.
