# Supplementary material for: Hetero‐trans‐β‐glucanase, an enzyme unique to Equisetum plants, functionalizes cellulose
Source: Plant J. 2015 Aug 25;83(5):753–69. doi: 10.1111/tpj.12935 (PMC4950035; doi:10.1111/tpj.12935)
Supplement: Supplementary file 5 — Figure S5. Inability of Pichia‐produced HTG to utilize cellohexaose as donor substrate. [file TPJ-83-753-s005.pptx]

## Slide 1
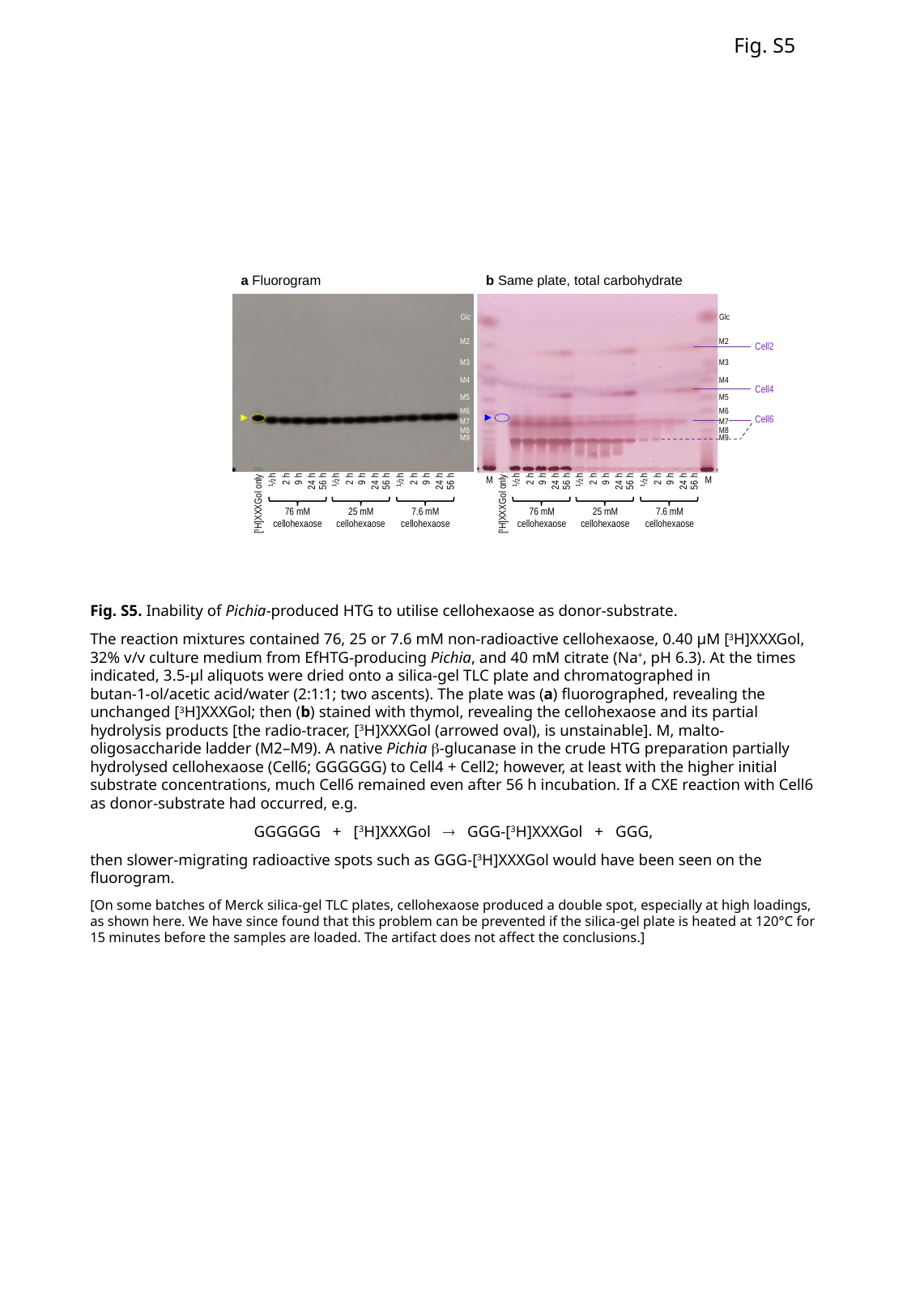

Fig. S5
a Fluorogram
b Same plate, total carbohydrate
Glc
Glc
Cell2
M2
M2
M3
M3
M4
M4
Cell4
M5
M5
M6
M6
Cell6
M7
M7
M8
M8
M9
M9
2 h
9 h
2 h
9 h
2 h
9 h
2 h
9 h
2 h
9 h
2 h
9 h
M
M
½ h
½ h
½ h
½ h
½ h
½ h
24 h
56 h
24 h
56 h
24 h
56 h
24 h
56 h
24 h
56 h
24 h
56 h
[3H]XXXGol only
[3H]XXXGol only
76 mM
cellohexaose
25 mM
cellohexaose
7.6 mM
cellohexaose
76 mM
cellohexaose
25 mM
cellohexaose
7.6 mM
cellohexaose
Fig. S5. Inability of Pichia-produced HTG to utilise cellohexaose as donor-substrate.
The reaction mixtures contained 76, 25 or 7.6 mM non-radioactive cellohexaose, 0.40 µM [3H]XXXGol, 32% v/v culture medium from EfHTG-producing Pichia, and 40 mM citrate (Na+, pH 6.3). At the times indicated, 3.5-µl aliquots were dried onto a silica-gel TLC plate and chromatographed in butan-1-ol/acetic acid/water (2:1:1; two ascents). The plate was (a) fluorographed, revealing the unchanged [3H]XXXGol; then (b) stained with thymol, revealing the cellohexaose and its partial hydrolysis products [the radio-tracer, [3H]XXXGol (arrowed oval), is unstainable]. M, malto-oligosaccharide ladder (M2–M9). A native Pichia β-glucanase in the crude HTG preparation partially hydrolysed cellohexaose (Cell6; GGGGGG) to Cell4 + Cell2; however, at least with the higher initial substrate concentrations, much Cell6 remained even after 56 h incubation. If a CXE reaction with Cell6 as donor-substrate had occurred, e.g.
GGGGGG + [3H]XXXGol  GGG-[3H]XXXGol + GGG,
then slower-migrating radioactive spots such as GGG-[3H]XXXGol would have been seen on the fluorogram.
[On some batches of Merck silica-gel TLC plates, cellohexaose produced a double spot, especially at high loadings, as shown here. We have since found that this problem can be prevented if the silica-gel plate is heated at 120°C for 15 minutes before the samples are loaded. The artifact does not affect the conclusions.]
