# Supplementary material for: Hetero‐trans‐β‐glucanase, an enzyme unique to Equisetum plants, functionalizes cellulose
Source: Plant J. 2015 Aug 25;83(5):753–69. doi: 10.1111/tpj.12935 (PMC4950035; doi:10.1111/tpj.12935)
Supplement: Supplementary file 6 — Figure S6. Effect of BSA on the activity of Pichia‐produced HTG with soluble and insoluble donor substrates. [file TPJ-83-753-s006.pptx]

## Slide 1
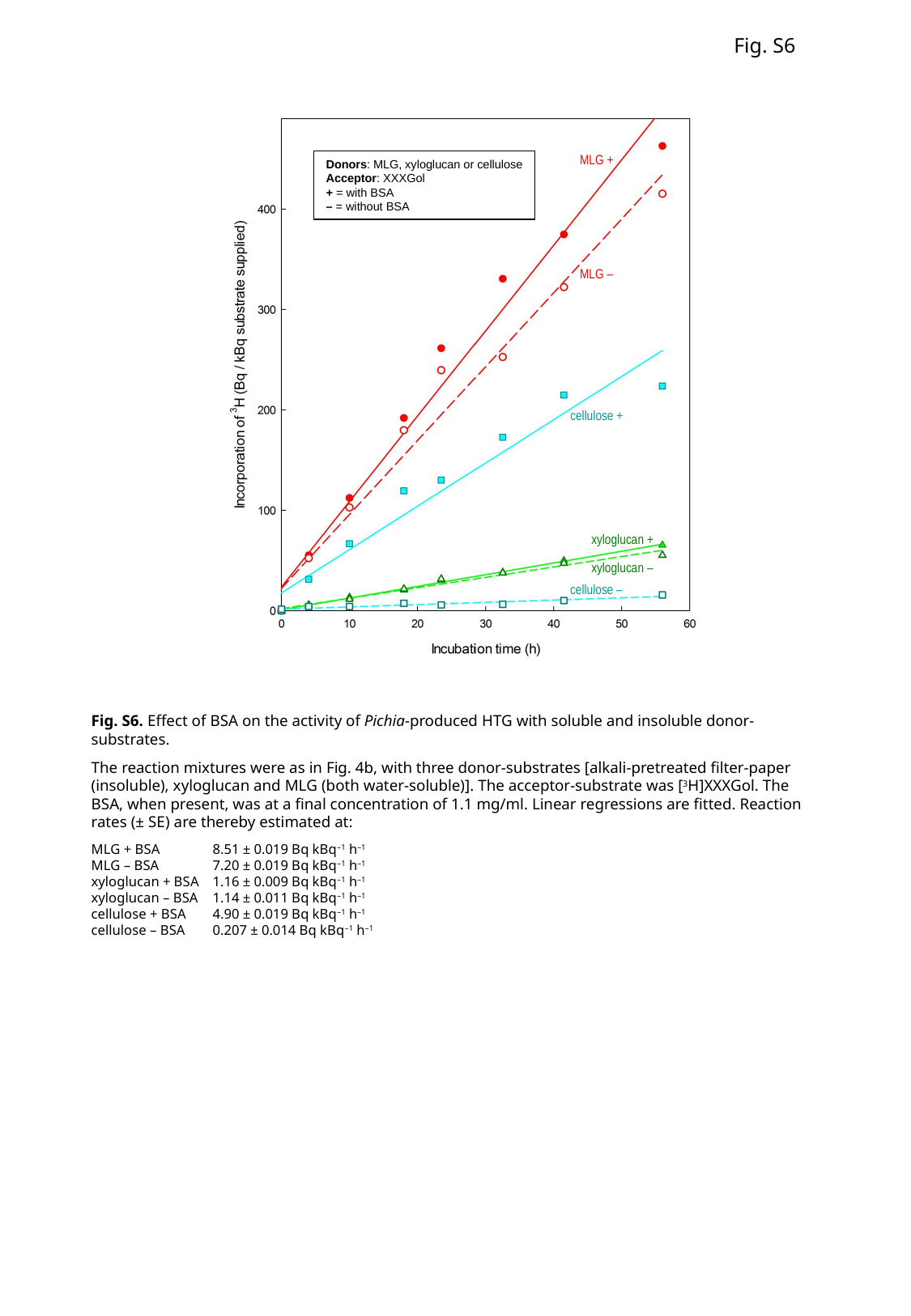

Fig. S6
Donors: MLG, xyloglucan or cellulose
Acceptor: XXXGol
+ = with BSA
– = without BSA
MLG +
MLG –
cellulose +
xyloglucan +
xyloglucan –
cellulose –
Fig. S6. Effect of BSA on the activity of Pichia-produced HTG with soluble and insoluble donor-substrates.
The reaction mixtures were as in Fig. 4b, with three donor-substrates [alkali-pretreated filter-paper (insoluble), xyloglucan and MLG (both water-soluble)]. The acceptor-substrate was [3H]XXXGol. The BSA, when present, was at a final concentration of 1.1 mg/ml. Linear regressions are fitted. Reaction rates (± SE) are thereby estimated at:
MLG + BSA	8.51 ± 0.019 Bq kBq–1 h–1
MLG – BSA	7.20 ± 0.019 Bq kBq–1 h–1
xyloglucan + BSA 	1.16 ± 0.009 Bq kBq–1 h–1
xyloglucan – BSA	1.14 ± 0.011 Bq kBq–1 h–1
cellulose + BSA	4.90 ± 0.019 Bq kBq–1 h–1
cellulose – BSA	0.207 ± 0.014 Bq kBq–1 h–1
