# Supplementary material for: Hetero‐trans‐β‐glucanase, an enzyme unique to Equisetum plants, functionalizes cellulose
Source: Plant J. 2015 Aug 25;83(5):753–69. doi: 10.1111/tpj.12935 (PMC4950035; doi:10.1111/tpj.12935)
Supplement: Supplementary file 7 — Table S1. Radiochemical characterization of cellulose–[3H]XXXGol generated by HTG extracted from E. fluviatile stems. [file TPJ-83-753-s007.docx]

**Supplementary TABLES**

**Table S1.** Radiochemical characterisation of cellulose–[^3^H]XXXGol generated by HTG extracted from *Equisetum fluviatile* stems

| **Enzyme-generated product** | **^3^H (Bq) obtained from** | |
| --- | --- | --- |
|  | **untreated paper (cellulose I)** | **NaOH‑pretreated paper (cellulose II)** |
| Radioactivity incorporated into paper and not washed out with water ‡ | * 35.7 ± 6.0 | * 55.3 ± 2.4 |
| **Of which:* | | |
| Washed out with 6 M NaOH / 0.66 M H_3_BO_3_ § | 5.0 ± 0.4 | 8.4 ± 1.1 |
| Inextractable in NaOH/borate (putative cellulose–[^3^H]XGO) § | 22.9 ± 3.6 | † 39.4 ± 4.8 |
| † *Of which:* | | |
| Solubilised in LiCl/DMA then re-precipitated with 6 M NaOH (cellulose–[^3^H]XGO) ¶ |  | 90.4% |
| Remaining in solution when 6 M NaOH added to LiCl/DMA ¶ |  | 9.6% |

‡ Triplicate 10-mg portions of Whatman No. 1 filter paper [either untreated (cellulose I) or converted to cellulose II in 6 M NaOH overnight then washed free of alkali and dried] were incubated with 100 µl of a reaction mixture containing [^3^H]XXXGol (2 kBq), HTG-enriched ammonium sulphate pellet from *Equisetum fluviatile* extract (Fig. S1a), and 0.3 M citrate buffer (Na^+^, pH 6.3). After 2 h, the reaction was stopped with formic acid (30 µl); the paper was exhaustively washed in water then assayed for incorporated ^3^H by scintillation counting. Data are mean ± SD, N=3.

§ To test the strength of ^3^H binding to the paper, we then washed the paper free of scintillation fluid with acetone, and incubated the paper at 25°C for 16 h in 6 M NaOH (containing 0.66 M H_3_BO_3_) at 50 ml per g paper. Soluble (cellulose oligomers and/or hemicelluloses) and remaining insoluble material (putative cellulose) were re-assayed for ^3^H. Data are mean ± SD, N=3.

¶ Putative ^3^H-labelled cellulose (CXE product; 40 mg, generated as above) was solvent-exchanged into acetone then dimethylacetamide (DMA; which had been dried over Sigma molecular sieve 4 Å for 5 d) and dissolved in 4 ml dry DMA containing 1.88 M LiCl at 20°C for 16 h. Another 4 ml of DMA was then added and the cellulose solution was added slowly (3.2 ml/h) to 80 ml of stirring 6 M NaOH; stirring was continued for a further 48 h. After centrifugation, the supernatant and pellet were separately neutralised with acetic acid and assayed for ^3^H by scintillation counting.
