# Supplementary material for: Hetero‐trans‐β‐glucanase, an enzyme unique to Equisetum plants, functionalizes cellulose
Source: Plant J. 2015 Aug 25;83(5):753–69. doi: 10.1111/tpj.12935 (PMC4950035; doi:10.1111/tpj.12935)
Supplement: Supplementary file 8 — Table S2. A four‐step strategy for purification of native Equisetum HTG. [file TPJ-83-753-s008.docx]

**Table S2.** A four-step strategy for purification of native *Equisetum* HTG

| **Step*** | **Protein (mg)** | **Total MXE activity (kBq h^–1^)** | **Specific activity (kBq h^−1^ mg^−1^ protein)** | **Yield of activity (%)** | **Fold purification** |
| --- | --- | --- | --- | --- | --- |
| crude extract | 75 | 263 | 3.51 | 100 | 1.0 |
| (NH_4_)_2_SO_4_ precipitation | 20 | 154 | 7.70 | 59 | 2.2 |
| Bio-Gel P-100 gel-permeation chromatography | 2.4 | 51.3 | 21.4 | 20 | 6.1 |
| Concanavalin-A lectin affinity chromatography | 0.68 | 39.3 | 57.8 | 15 | 16 |
| Isoelectric focusing | 0.11 | 26.5 | 241 | 10 | 69 |

*We purified HTG from a crude enzyme extract from late-season *E. fluviatile* lateral shoots using a four-step protocol and by tracking the presence of MXE activity. All purification steps were performed at 5°C. Total *E. fluviatile* extract (100 ml) was precipitated in 10–60% (v/v) saturated ammonium sulphate (in 10% ‘cuts’) followed by centrifugation. Each cut was redissolved in 25 ml 10 mM citrate (Na^+^, pH 6.1) containing 0.05% Triton X-100. In this experiment, the 30–40% cut contained the highest activity, 6 ml of which was fractionated by gel-permeation chromatography on Bio-Gel P-100. MXE-rich fractions were then further fractionated on a 1.8-ml bed-volume column of concanavalin A–agarose; MXE activity was eluted from the column by 640 mM methyl α-mannopyranoside. MXE-rich concanavalin A fractions were fractionated by isoelectric focusing, after which MXE activity was found in a region of pH ≈ 4.0. A 5-µl portion of the enzyme solution obtained after each step was assayed for MXE activity in a 10-µl reaction mixture containing 5 mg/ml MLG and 1 kBq [^3^H]XXXGol at 25°C for 4 h. ‘Total MXE activity’ is calculated according to the total volume from which the 5 µl was sampled.
