# Supplementary material for: Hetero‐trans‐β‐glucanase, an enzyme unique to Equisetum plants, functionalizes cellulose
Source: Plant J. 2015 Aug 25;83(5):753–69. doi: 10.1111/tpj.12935 (PMC4950035; doi:10.1111/tpj.12935)
Supplement: Supplementary file 9 — Table S3. Modelled interactions between xyloglucan and the active site in Populus XTH (PttXET) (Johansson et al., 2004), Tropaeolum XTH (TmNXG1) (Mark et al., 2009) and Equisetum HTG (present work). [file TPJ-83-753-s009.docx]

**Table S3.** Modelled interactions between xyloglucan and the active site in *Populus* XTH (PttXET) (Johansson *et al.*, 2004), *Tropaeolum* XTH (TmNXG1) (Mark *et al.*, 2009) and *Equisetum* HTG (present work).

Subsite –1 is defined as that occupied by the donor-substrate glucose residue whose glycosidic bond is cleaved during transglycosylation; positive subsites will be occupied by the incoming acceptor substrate. The xyloglucan adopted for modelling was non-galactosylated. Unique features of HTG are highlighted in yellow. In all cases, these features of EfHTG are shared with Ed39394 and Eh12712 (the putative HTGs of other *Equisetum* spp.; see Fig. S3b). Other residues that structural analysis suggests may also contribute to differences in substrate specificity are highlighted in green.

| **Subsite sugar** | **Model** | **Ligand group** | **Protein group** | **Interaction type** |
| --- | --- | --- | --- | --- |
| –4 Glc | EfHTG | O-2 | Pro10 backbone O | H-bond |
|  | PttXET | — | — | — |
|  | TmNXG1 | — | — | — |
| –4 Glc | EfHTG | O-4 | Ser31 sidechain | H-bond |
|  | PttXET | O-4 | Thr41 sidechain | H-bond |
|  | TmNXG1 | O-4 | Ser47 sidechain | H-bond |
| –3 Glc | EfHTG | Pyranose ring | Pro10 sidechain | Hydrophobic |
|  | PttXET | Pyranose ring | Trp19 sidechain | Stacking |
|  | TmNXG1 | Pyranose ring | Trp27 sidechain | Stacking |
| –3 Xyl* | EfHTG | — | — | — |
|  | PttXET | O-5 | Trp19 sidechain | H-bond |
|  | TmNXG1 | O-3 and O-4,  O-3 | Asn85 sidechain,  Gln86 sidechain | H-bonds,  H-bond |
| –3 Xyl | EfHTG | — | — | — |
|  | PttXET | — | — | — |
|  | TmNXG1 | Pyranose ring | Leu26 sidechain | Hydrophobic |
| –2 Glc | EfHTG | O-2 | Ser162 sidechain | H-bond |
|  | PttXET | O-2 | Ser172 sidechain | H-bond |
|  | TmNXG1 | O-2 | Ser178 sidechain | H-bond |
| –2 Glc | HTG | Pyranose ring | Trp164 sidechain | Stacking |
|  | PTTXET | Pyranose ring | Trp174 sidechain | Stacking |
|  | NXG1 | Pyranose ring | Trp180 sidechain | Stacking |
| –2 Glc | HTG | O-3 | Ser34 sidechain | H-bond |
|  | PTTXET | — | — | — |
|  | NXG1 | — | — | — |
| –2 Glc | HTG | O-2 | Thr63 sidechain | H-bond |
|  | PTTXET | — | — | — |
|  | NXG1 | O-2 | Ser79 side chain | H-bond |
| –1 Glc † | EfHTG | O-6 | Glu79 sidechain | H-bond |
|  | PttXET | O-3 | Glu89 sidechain | H-bond |
|  | TmNXG1 | O-3 | Glu98 sidechain | H-bond |
| –1 Glc | EfHTG | Pyranose ring | Tyr65 sidechain | Stacking |
|  | PttXET | Pyranose ring | Tyr75 sidechain | Stacking |
|  | TmNXG1 | Pyranose ring | Tyr81 sidechain | Stacking |
| +1 Glc | EfHTG | O-3 | Asp77 sidechain | H-bond |
|  | PttXET | O-3 | Asp77 sidechain | H-bond |
|  | TmNXG1 | O-3 | Asp87 sidechain | H-bond |
| +1 Glc | EfHTG | Pyranose ring | Trp169 sidechain | Stacking |
|  | PttXET | Pyranose ring | Trp179 sidechain | Stacking |
|  | TmNXG1 | Pyranose ring | Trp185 sidechain | Stacking |
| +1 Glc | EfHTG | O-2 | His92 sidechain | H-bond |
|  | PttXET | O-2 | Gln102 sidechain | H-bond |
|  | TmNXG1 | O-2 | Gln111 sidechain | H-bond |
| **Subsite sugar** | **Model** | **Ligand group** | **Protein group** | **Interaction type** |
| +1 Glc | EfHTG | O-3 | Asn94 sidechain | H-bond |
|  | PttXET | O-3 | Asn104 sidechain | H-bond |
|  | TmNXG1 | O-3 | Asn113 sidechain | H-bond |
| +1 Glc | EfHTG | O-2 | Glu79 sidechain | H-bond |
|  | PttXET | O-3 | Glu89 sidechain | H-bond |
|  | TmNXG1 | O-3 | Glu98 sidechain | H-bond |
| +1 Xyl | EfHTG | O-2 | Trp169 sidechain | H-bond |
|  | PttXET | O-2 | Trp179 sidechain | H-bond |
|  | TmNXG1 | O-2 | Trp185 sidechain | H-bond |
| +1 Xyl | EfHTG | — | — | — |
|  | PttXET | O-2 and O-3 | Asp178 sidechain | H-bonds |
|  | TmNXG1 | — | — | — |
| +2 Glc | EfHTG | Pyranose ring | Trp169 sidechain | Stacking |
|  | PttXET | Pyranose ring | Trp179 sidechain | Stacking |
|  | TmNXG1 | Pyranose ring | Trp185 sidechain | Stacking |
| +2 Xyl | EfHTG | Pyranose ring | Tyr237 sidechain | Stacking |
|  | PttXET | Pyranose ring | Tyr250 sidechain | Stacking |
|  | TmNXG1 | Pyranose ring | Tyr250 sidechain | Stacking |
| +2 Xyl | EfHTG | Pyranose ring | Gln106 sidechain | Hydrophobic (weak) |
|  | PttXET | Pyranose ring | Arg116 sidechain | Hydrophobic |
|  | TmNXG1 | Pyranose ring | Arg125 sidechain | Hydrophobic |
| +2 Xyl | EfHTG | — | — | — ‡ |
|  | PttXET | Pyranose ring | Arg258 sidechain | Hydrophobic |
|  | TmNXG1 | Pyranose ring | Arg258 sidechain | Hydrophobic |
| +3 Glc | EfHTG |  |  |  |
|  | PttXET |  |  |  |
|  | TmNXG1 | O-2 | Asn189 sidechain | H-bond |

* In TmNXG1, Asn85 H-bonds to the O3 of the –3 xylose. The equivalent residue in PttXET is Gln79, and in the XET crystal structure (1UMZ) it is positioned too far from the xylose to interact with it. However, this crystal structure lacks any substrate in the minus subsites. There is evidence that large conformation changes take place upon substrate binding [for example, in the PttXET structure Trp174 (Trp164 in EfHTG and Trp180 in TmNXG1) and Trp19 (Pro10 in EfHTG and Trp27 in TmNXG1) are in apo conformations which flip upon substrate binding], which may bring this residue close enough to hydrogen bond to the xylose when it is present. Therefore it is possible that Gln79 in PttXET makes the same interaction with the –3 xylose as Asn85 in TmNXG1 does, even if the models are unable to capture this. In EfHTG the residue at this position is Asp69, which is too far to form a hydrogen bond with the –3 xylose; and, unlike PttXET, this residue could not undergo a conformation change to bring it into closer proximity to the substrate because of the bulky sidechain of Phe67 (Ser in both TmNXG1 and PttXET) occupying the necessary space.

† No interactions are made between any modelled protein and the –2 xylose.

‡ EfHTG has Leu245, and Ed39394 and Eh12712 (the putative homologues in other *Equisetum* spp.; see Fig. S3b) both have a corresponding Leu. In contrast, all standard XTHs have Arg (or Lys): this Arg forms a salt bridge with a Glu that is predicted to pull both flat, forming a hydrophobic platform that interacts with the pyranose ring of the +2 Xyl.
