# Supplementary material for: Hetero‐trans‐β‐glucanase, an enzyme unique to Equisetum plants, functionalizes cellulose
Source: Plant J. 2015 Aug 25;83(5):753–69. doi: 10.1111/tpj.12935 (PMC4950035; doi:10.1111/tpj.12935)
Supplement: Supplementary file 10 — Methods S1. Mass spectrometry, gene amplification, cloning, and heterologous protein production. [file TPJ-83-753-s010.docx]

**Supplementary Experimental Methods S1.**

**Mass spectrometry, gene amplification, cloning, and heterologous protein production**

**Mass spectrometry**

Coomassie-stained bands were tightly cut from SDS gels and incubated 2× in 300 µl 200 mM NH_4_HCO_3_, 50% (v/v) acetonitrile at 25°C for 30 min. Each excised band was incubated in 300 µl 20 mM dithiothreitol, 200 mM NH_4_HCO_3_, 50% acetonitrile at 25°C for 1 h, washed 3× in 300 µl 200 mM NH_4_HCO_3_, 50% acetonitrile, and incubated in 100 µl freshly made 50 mM iodoacetamide, 200 mM NH_4_HCO_3_, 50% acetonitrile at 25°C in the dark for 20 min. Bands were washed 3× in 500 µl 20 mM NH_4_HCO_3_, 50% acetonitrile, then cut into smaller (2 × 1 mm) pieces, centrifuged, covered with acetonitrile for 5 min, then dried until the gel pieces turned white. Gel pieces were incubated in 30 µl 13.3 µg/ml trypsin (Promega) in 50 mM NH_4_HCO_3_ at 4°C until the gel swelled, then at 32°C for 16 h. Internal calibration peaks (trypsin autolysis products) were provided by a trypsin-only blank. Samples were then sonicated for 10 min and solubilised peptides collected.

For MALDI–TOF MS analysis, 0.5-µl aliquots of digests were mixed with 0.5 µl 10 mg/ml α-cyano-4-hydroxycinnamic acid (Sigma) in 50% acetonitrile containing 3 mM trifluoroacetic acid and allowed to dry. Samples were analysed on a Voyager DE-STR MALDI–TOF MS (Applied Biosystems).

For LC–MS analysis, 5 µl sample (de-salted on a C18 StageTip) (Rappsilber *et al.*, 2003) was loaded onto an HTC PAL autosampler (CTC Analytics, Switzerland) in series with an Agilent 1200 Series HPLC with a PicoTip emitter (FS 360-100-8-N-20-C12, New Objective). The emitter column was packed with Reprosil-Pur C18-AQ 3µm (Dr Maisch GmbH) to a length of 6.5–7.5 cm and equilibrated with solvent A (acetic acid/acetonitrile/water, 1:10:189 v/v); the sample was equilibrated on the column at 0.7 µl/min with 100% A (0–8 min) and eluted at 0.3 µl/min with linear gradients of 0–60% B in A (where B is acetic acid/acetonitrile, 1:199, v/v; 8–38 min), 60–80% B (38–45 min), and 100% B (45–68 min). Data acquisition was controlled by Xcalibur software (ThermoScientific).

Processed spectra were searched against the NCBI non-redundant database and an *E. fluviatile* transcriptome database using in-house licensed MASCOT software.

**Gene amplification and cloning**

Rapid amplification of cDNA ends (RACE) yielded full-length EfHTG cDNA sequences. Templates for 5´-RACE were created by reverse transcription in 1.5 µM dT_30_ primer, 1.5 µM of Smart II oligonucleotide (5´-AAGCAGTGGTAACAACGCAGAGTACGCGGG-3´), 0.5 mM of each dNTP, 50 mM Tris (Cl^−^, pH 8.3), 75 mM KCl, 3 mM MgCl_2_, 2 mM MnCl_2_, 10 mM dithiothreitol, 0.25 µg/µl BSA, 50 ng/µl *Equisetum* total RNA, 10 units/µl Superscript II reverse transcriptase (Life Technologies) and 1 unit/µl RNasin (Promega). The solution of RNA and oligonucleotides was incubated at 80°C (5 min) then 0°C before addition of other components and incubation at 25°C (10 min) and 42°C (1 h). cDNA was purified with a QIAquick PCR purification kit (Qiagen) according to supplier’s instructions. cDNA templates for 3´-RACE were produced similarly, except that Q_T_ (5´-CCAGTGAGCAGAGTGACGAGGACTCGAGCTCAAGCT_17_VN-3´) was used to prime cDNA synthesis and MnCl_2_ omitted.

5´-cDNAs were amplified by polymerase chain reaction (PCR) with high-fidelity Phusion DNA polymerase (New England Biolabs) in a mixture containing 400-fold diluted purified cDNA, 1× Phusion buffer, 0.2 mM each dNTP, 0.2 µM of an EfHTG-specific primer, 40 nM UPML primer [complementary to part of the Smart II oligonucleotide added to the 3´ end of cDNAs during synthesis (5´-CTAATACGACTCACTATAGGGCAAGCAGTGGTATCAACGCAGAGT-3´)] and 200 nM primer UPMS nested within UPML (5´-CTAATACGACTCACTATAGGGC-3´). 3´-RACE amplification used an EfHTG-specific primer and Q_O_ (5´-CCAGTGAGCAGAGTGACG-3´), corresponding to the 5´-end of Q_T_.

PCR product lengths were estimated by agarose gel electrophoresis and ligated into pJET1.2 vector (Fermentas), which was used to transform *E. coli* strain DH5α. Bacterial colonies were screened by PCR, with primers flanking the pJET1.2 cloning site and PCR products of appropriate size were used directly in Sanger DNA sequencing, carried out by Edinburgh Genomics.

To express EfHTG as a fusion protein from the *Pichia* pPICZαA vector, we amplified cDNA with Phusion polymerase and primers 5´-GAATTCGGTTTCTATGGGGACTTTCAG-3´, to introduce a 5´ *Eco* RI site immediately upstream of the sequence encoding the putative N-terminus of mature EfHTG and 5´-TCTAGATAGAAACCACGGTTTGAGCATT-3´ to remove the EfHTG stop codon and introduce an *Xba* I site. To confirm absence of PCR-induced mutations, we cloned the PCR product in pJET1.2 and sequenced it. It was then excised from the vector by complete digestion with *Xba* I and partial digestion with *Eco* RI (EfHTG contains an internal *Eco* RI site), gel-purified and ligated into pPICZαA.

**Recombinant EfHTG production in *Pichia pastoris***

*E. coli* carrying recombinant pPICZαA was selected with 40 µg/ml zeocin (InVitrogen) on low-salt (65 mM NaCl) L-agar at pH 7.5. Plasmids were linearised by digestion with *Sac* I and used to transform *Pichia pastoris* strain SMD1168H by electroporation (1.6 kV, 1-mm cuvette) with selection on low-salt L-agar containing 100 mg/l zeocin. Positive colonies were confirmed by PCR with EfHTG-specific primers and grown overnight in low-salt L-B medium supplemented with 100 mg/l zeocin, 1% (w/v) glycerol and 0.4 mg/l biotin. Cells were recovered by centrifugation and re-suspended in expression medium (as above, but with glycerol replaced by 10% methanol). After 24 h, cell-free medium was assayed for transglucanase activities.

Recombinant EfHTG carried a C-terminal His_6_ tag allowing purification by affinity chromatography on a 2-ml Chelating Sepharose column (GE Healthcare Ltd) saturated with Ni^2+^. For enzyme preparations used in assaying substrate specificity (Figs 4g and 5c), the secreted *Pichia* proteins were bound to the column in buffer A (20 mM Na_2_HPO_4_, 0.75 M NaCl, 0.05% Triton X-100 at pH 7.0) and eluted with 50 mM imidazole in the same buffer. A PD-10 desalting column (GE Healthcare) was then used for transferring the protein into buffer B (20 mM citrate, pH 6.3, containing 0.05 % Triton X-100 but no NaCl) with 0.5% (w/v) chlorobutanol. For enzyme preparations used in western blotting (Fig. 3), *Pichia* culture filtrate (3.5 ml, mixed with 3.5 ml buffer A) was applied. Proteins were then eluted with, sequentially: 5 ml A, 3 ml A adjusted to 1.5 M NaCl, 2 ml A, 6 ml of a gradient from buffer A to buffer B, and finally buffers C (5 mM citrate and 5 mM formate, pH adjusted to values between 6.1 and 3.7, in steps of 0.2 pH units); 1-ml fractions were collected.

*Pichia*-produced HTG, partially purified by the second method, was subjected to SDS–PAGE on a 12% gel, blotted onto a nitrocellulose membrane (<http://www.bio-rad.com/webroot/web/pdf/lsr/literature/M1703940.pdf>), and detected with a mouse anti-His-tag antibody (1:2000; Pierce, Thermo Scientific) followed by horse anti-mouse IgG secondary antibody (1:1000; horseradish peroxidase-conjugated). The membrane was incubated in chemiluminescent substrate (WestPico, Thermo Scientific) and the signal captured on X-ray film.
