# Supplementary material for: Hetero‐trans‐β‐glucanase, an enzyme unique to Equisetum plants, functionalizes cellulose
Source: Plant J. 2015 Aug 25;83(5):753–69. doi: 10.1111/tpj.12935 (PMC4950035; doi:10.1111/tpj.12935)
Supplement: Supplementary file 11 [file TPJ-83-753-s011.docx]

Supporting Information Legends

**Fig. S1.** Fractionation of native *Equisetum* HTG.

(a) MXE and XET activities co-elute during cation-exchange chromatography. *E. fluviatile* protein, precipitated with 20%-saturated ammonium sulphate (main text, Fig. 1g), was dialysed against pH 3.7 buffer (10 mM each of citrate and formate, Na^+^, pH 3.7), then applied to a column of Sulphoethyl-Sephadex C50 (bed volume 5 ml; pre-equilibrated in the same buffer), followed by 10-ml portions of the same buffer (supplemented with 0.05% Triton X-100) at each pH (3.7–5.1); 2.5-ml fractions were collected. MXE and XET activity in 20-µl aliquots were assayed in excess 75 mM citrate (Na^+^, pH 6.3) with [^3^H]XXXGol (1 kBq) as acceptor-substrate and MLG or xyloglucan (2.4 mg/ml) as donor-substrate for 3 h.

(b) MXE and XET activities co-elute during gel-permeation chromatography. *E. fluviatile* protein, as in (A), was applied to a column of Bio-Gel P-100 and eluted at 14 ml h^−1^; 2-ml fractions were assayed for *A*_280_ and for MXE and XET activity as in (a). The *A*_280_ of the *Equisetum* sample was measured in a suitable dilution of the eluate. Markers (5–40-MDa dextran; cytochrome *c*_551_, M_r_ ≈ 1×10^4^; and CoCl_2_) were run through the same column under identical conditions and recorded by absorbance at 600, 410 and 510 nm respectively.

(c) Protein content and MXE and XET activities of *E. fluviatile* extracts progressively precipitated with increasing (NH_4_)_2_SO_4_. Data are per 300 mg tissue. Numbers above bars: specific activity (Bq kBq^−1^ h^−1^ mg protein^−1^).

(d) Electrophoresis of proteins in the 20%-saturated (NH_4_)_2_SO_4_ cut. Three identical lanes of the gel were applied to test papers [similar to those used in (d*–*f)], documenting MXE, XET and CXE activities. CB = gel stained for protein with Coomassie Blue. ▶ = approximate co-migration of the three transglycanase activities.

**Fig. S2.** Mass spectra of tryptic peptides of *Equisetum fluviatile* HTG.

(a) MALDI–ToF mass spectrum of intact tryptic peptides from IEF/SDS-purified HTG (see Fig, 2e). The two peaks labelled in blue have m/z values corresponding to tryptic peptides predicted from an XTH-related nucleotide sequence found in the *Equisetum* transcriptome.

(b) LC–MS/MS fragmentation spectra of the two tryptic peptides highlighted in (a), whose doubly-charged parent ions had *m*/*z* 482.26 and 774.91 respectively. Each spectrum identifies a different partial XTH-homologue mRNA found in the *Equisetum* transcriptome.

(c) The corresponding putative amino acid sequences taken from a MASCOT search; the *m*/*z* values of detected positive ion fragments are in red. The ‘b’ and ‘y’ ions are singly charged fragments (molecule + 1 H^+^) produced by fragmentation from the N- and C-terminus respectively; ‘b^++^’ and ‘y^++^’ ions are the corresponding doubly charged fragments (molecule + 2 H^+^). The y* ions are y ions with loss of water. Fragmentation spectra matching two further *in-silico* peptides (ENYLK and ENYLKYDYCYDTK) from HTG partial transcriptome sequences were also identified.

† For explanation, see http://www.matrixscience.com/help/interpretation_help.html

‡ For explanation, see http://www.matrixscience.com/help/scoring_help.html

**Fig. S3.** Deduced sequence of *Equisetum fluviatile* HTG, compared with several other family-GH16b proteins.^a^

(a) Sequence of *Equisetum fluviatile* HTG; residues numbered contiguously after removal of the predicted signal peptide (which is MLGLVFGMLVIMLASPKLAMA). The tryptic peptides noted in Fig. S2 are shown in colour.

(b) HTG sequence, with gaps to maximise alignment with other family-GH16b sequences. The plant proteins are from Ef, *Equisetum fluviatile* (present work); Ed, *Equisetum diffusum* and Eh, *Equisetum hyemale* (hitherto non-annotated sequences from www.onekp.com); and At, *Arabidopsis thaliana* (33 XTH sequences^b^). The lichenase A sequence is from (A) *Bacillus subtilis* (accession no. AAB05759), lichenase B from *B. subtilis* (WP003244531), and lichenase C from *B. licheniformis* (P27051). All sequences shown are after removal of the predicted signal peptide. Arrows indicate key residues at which the three *Equisetum* HTG sequences differ from all others (cf. main text Fig. 6).

^a^ Strohmeier M *et al.* (2004) Molecular modeling of family GH16 glycoside hydrolases: potential roles for xyloglucan transglucosylases/hydrolases in cell wall modification in the Poaceae. *Protein Sci* **13**: 3200–3213.

^b^ Yokoyama R, Nishitani K (2001) A comprehensive expression analysis of all members of a gene family encoding cell-wall enzymes allowed us to predict *cis-regulatory* regions involved in cell-wall construction in specific organs of Arabidopsis. *Plant Cell Physiol* **42**: 1025–1033.

**Fig. S4.** Rooted cladogram showing the relationship of HTG to other GH16b subfamily members

Inferred amino acid sequences were aligned with MUSCLE ^a^ in MEGA6 ^b^, and manually trimmed to remove likely N-terminal leaders that showed little homology. Relationships were inferred by maximum likelihood in MEGA6, by use of the JTT model of amino acid substitutions and deletion of positions with gaps in more than 25% of sequences. Nodes recovered in at least 50% of bootstrap replicates are marked with dots. Protein clades and the gene products of *Arabidopsis thaliana* (At) and rice (Os) are labelled as in ref ^c^. The tree is rooted on the “Ancestral” clade identified as basal among XTHs ^d^. Sequences from *Selaginella moellendorffii* (Sm) were inferred from genomic sequences from www.phytozome.net and those of the ferns *Pteridium aquilinum* (Pa) and *Adiantum capillus-veneris* (Ac) from EST sequences from http://acest.biol.se.tmu.ac.jp/blast/. *Equisetum fluviatile* (Ef) proteins were translated from cDNA sequences as part of this study. Likely orthologs of EfHTG from *E. hyemale* (Eh12712) and *E. diffusum* (Ed39394) were obtained from www.onekp.com. EfHTG is highlighted with an asterisk, related *E. fluviatile* proteins that showed only XET activity when produced in *Pichia* with open squares and the *Tropaeolum majus* (Tm) and *Populus* *tremuloides* (Ptt) proteins used in structure modelling (Fig. 6) with arrowheads.

^a^ Edgar RC (2004) MUSCLE: multiple sequence alignment with high accuracy and high throughput. *Nucl Acids Res* **32**: 1792–1792.

^b^ Tamura K, Stecher G, Peterson D, Filipski A, Kumar S (2013) MEGA6: Molecular Evolutionary Genetics Analysis Version 6.0. *Molecular Biology and Evolution* **30**: 2725–2729.

^c^ Eklöf JM, Shojania S, Okon M, McIntosh LP, Brumer H (2013) Structure–function analysis of a broad specificity Populus trichocarpa endo-β-glucanase reveals an evolutionary link between bacterial licheninases and plant XTH gene products. *J Biol Chem* **288**: 15786–15799.

^d^ Baumann MJ *et al.* (2007) Structural evidence for the evolution of xyloglucanase activity from xyloglucan endo-transglycosylases: biological implications for cell wall metabolism. *Plant Cell* **19**: 1947–1963.

**Fig. S5.** Inability of *Pichia*-produced HTG to utilise cellohexaose as donor-substrate.

The reaction mixtures contained 76, 25 or 7.6 mM non-radioactive cellohexaose, 0.40 µM [^3^H]XXXGol, 32% v/v culture medium from EfHTG-producing *Pichia*, and 40 mM citrate (Na^+^, pH 6.3). At the times indicated, 3.5-µl aliquots were dried onto a silica-gel TLC plate and chromatographed in butan-1-ol/acetic acid/water (2:1:1; two ascents). The plate was (**a**) fluorographed, revealing the unchanged [^3^H]XXXGol; then (**b**) stained with thymol, revealing the cellohexaose and its partial hydrolysis products [the radio-tracer, [^3^H]XXXGol (arrowed oval), is unstainable]. M, malto-oligosaccharide ladder (M2–M9). A native *Pichia* β-glucanase in the crude HTG preparation partially hydrolysed cellohexaose (Cell6; GGGGGG) to Cell4 + Cell2; however, at least with the higher initial substrate concentrations, much Cell6 remained even after 56 h incubation. If a CXE reaction with Cell6 as donor-substrate had occurred, e.g.

GGGGGG + [^3^H]XXXGol → GGG-[^3^H]XXXGol + GGG,

then slower-migrating radioactive spots such as GGG-[^3^H]XXXGol would have been seen on the fluorogram.

[On some batches of Merck silica-gel TLC plates, cellohexaose produced a double spot, especially at high loadings, as shown here. We have since found that this problem can be prevented if the silica-gel plate is heated at 120°C for 15 minutes before the samples are loaded. The artifact does not affect the conclusions.]

**Fig. S6.** Effect of BSA on the activity of *Pichia*-produced HTG with soluble and insoluble donor-substrates.

The reaction mixtures were as in Fig. 4b, with three donor-substrates [alkali-pretreated filter-paper (insoluble), xyloglucan and MLG (both water-soluble)]. The acceptor-substrate was [^3^H]XXXGol. The BSA, when present, was at a final concentration of 1.1 mg/ml. Linear regressions are fitted. Reaction rates (± SE) are thereby estimated at:

MLG + BSA 8.51 ± 0.019 Bq kBq^–1^ h^–1^

MLG – BSA 7.20 ± 0.019 Bq kBq^–1^ h^–1^

xyloglucan + BSA 1.16 ± 0.009 Bq kBq^–1^ h^–1^

xyloglucan – BSA 1.14 ± 0.011 Bq kBq^–1^ h^–1^

cellulose + BSA 4.90 ± 0.019 Bq kBq^–1^ h^–1^

cellulose – BSA 0.207 ± 0.014 Bq kBq^–1^ h^–1^

**Table S1.** Radiochemical characterisation of cellulose–[^3^H]XXXGol generated by HTG extracted from *Equisetum fluviatile* stems

‡ Triplicate 10-mg portions of Whatman No. 1 filter paper [either untreated (cellulose I) or converted to cellulose II in 6 M NaOH overnight then washed free of alkali and dried] were incubated with 100 µl of a reaction mixture containing [^3^H]XXXGol (2 kBq), HTG-enriched ammonium sulphate pellet from *Equisetum fluviatile* extract (Fig. S1a), and 0.3 M citrate buffer (Na^+^, pH 6.3). After 2 h, the reaction was stopped with formic acid (30 µl); the paper was exhaustively washed in water then assayed for incorporated ^3^H by scintillation counting. Data are mean ± SD, N=3.

§ To test the strength of ^3^H binding to the paper, we then washed the paper free of scintillation fluid with acetone, and incubated the paper at 25°C for 16 h in 6 M NaOH (containing 0.66 M H_3_BO_3_) at 50 ml per g paper. Soluble (cellulose oligomers and/or hemicelluloses) and remaining insoluble material (putative cellulose) were re-assayed for ^3^H. Data are mean ± SD, N=3.

¶ Putative ^3^H-labelled cellulose (CXE product; 40 mg, generated as above) was solvent-exchanged into acetone then dimethylacetamide (DMA; which had been dried over Sigma molecular sieve 4 Å for 5 d) and dissolved in 4 ml dry DMA containing 1.88 M LiCl at 20°C for 16 h. Another 4 ml of DMA was then added and the cellulose solution was added slowly (3.2 ml/h) to 80 ml of stirring 6 M NaOH; stirring was continued for a further 48 h. After centrifugation, the supernatant and pellet were separately neutralised with acetic acid and assayed for ^3^H by scintillation counting.

**Table S2.** A four-step strategy for purification of native *Equisetum* HTG

*We purified HTG from a crude enzyme extract from late-season *E. fluviatile* lateral shoots using a four-step protocol and by tracking the presence of MXE activity. All purification steps were performed at 5°C. Total *E. fluviatile* extract (100 ml) was precipitated in 10–60% (v/v) saturated ammonium sulphate (in 10% ‘cuts’) followed by centrifugation. Each cut was redissolved in 25 ml 10 mM citrate (Na^+^, pH 6.1) containing 0.05% Triton X-100. In this experiment, the 30–40% cut contained the highest activity, 6 ml of which was fractionated by gel-permeation chromatography on Bio-Gel P-100. MXE-rich fractions were then further fractionated on a 1.8-ml bed-volume column of concanavalin A–agarose; MXE activity was eluted from the column by 640 mM methyl α-mannopyranoside. MXE-rich concanavalin A fractions were fractionated by isoelectric focusing, after which MXE activity was found in a region of pH ≈ 4.0. A 5-µl portion of the enzyme solution obtained after each step was assayed for MXE activity in a 10-µl reaction mixture containing 5 mg/ml MLG and 1 kBq [^3^H]XXXGol at 25°C for 4 h. ‘Total MXE activity’ is calculated according to the total volume from which the 5 µl was sampled.

**Table S3.** Modelled interactions between xyloglucan and the active site in *Populus* XTH (PttXET) (Johansson *et al.*, 2004), *Tropaeolum* XTH (TmNXG1) (Mark *et al.*, 2009) and *Equisetum* HTG (present work).

Subsite –1 is defined as that occupied by the donor-substrate glucose residue whose glycosidic bond is cleaved during transglycosylation; positive subsites will be occupied by the incoming acceptor substrate. The xyloglucan adopted for modelling was non-galactosylated. Unique features of HTG are highlighted in yellow. In all cases, these features of EfHTG are shared with Ed39394 and Eh12712 (the putative HTGs of other *Equisetum* spp.; see Fig. S3b). Other residues that structural analysis suggests may also contribute to differences in substrate specificity are highlighted in green.

* In TmNXG1, Asn85 H-bonds to the O3 of the –3 xylose. The equivalent residue in PttXET is Gln79, and in the XET crystal structure (1UMZ) it is positioned too far from the xylose to interact with it. However, this crystal structure lacks any substrate in the minus subsites. There is evidence that large conformation changes take place upon substrate binding [for example, in the PttXET structure Trp174 (Trp164 in EfHTG and Trp180 in TmNXG1) and Trp19 (Pro10 in EfHTG and Trp27 in TmNXG1) are in apo conformations which flip upon substrate binding], which may bring this residue close enough to hydrogen bond to the xylose when it is present. Therefore it is possible that Gln79 in PttXET makes the same interaction with the –3 xylose as Asn85 in TmNXG1 does, even if the models are unable to capture this. In EfHTG the residue at this position is Asp69, which is too far to form a hydrogen bond with the –3 xylose; and, unlike PttXET, this residue could not undergo a conformation change to bring it into closer proximity to the substrate because of the bulky sidechain of Phe67 (Ser in both TmNXG1 and PttXET) occupying the necessary space.

† No interactions are made between any modelled protein and the –2 xylose.

‡ EfHTG has Leu245, and Ed39394 and Eh12712 (the putative homologues in other *Equisetum* spp.; see Fig. S3b) both have a corresponding Leu. In contrast, all standard XTHs have Arg (or Lys): this Arg forms a salt bridge with a Glu that is predicted to pull both flat, forming a hydrophobic platform that interacts with the pyranose ring of the +2 Xyl.
